# PhDHS Is Involved in Chloroplast Development in Petunia

**DOI:** 10.3389/fpls.2019.00284

**Published:** 2019-03-13

**Authors:** Juanxu Liu, Xinlei Chang, Beibei Ding, Shan Zhong, Li Peng, Qian Wei, Jie Meng, Yixun Yu

**Affiliations:** Guangdong Key Laboratory for Innovative Development and Utilization of Forest Plant Germplasm, College of Forestry and Landscape Architecture, South China Agricultural University, Guangzhou, China

**Keywords:** deoxyhypusine synthase, chloroplast development, sectored chlorotic leaf, photosynthesis, petunia

## Abstract

Deoxyhypusine synthase (DHS) is encoded by a nuclear gene and is the key enzyme involved in the post-translational activation of the eukaryotic translation initiation factor eIF5A. DHS plays important roles in plant growth and development. To gain a better understanding of DHS, the petunia (*Petunia hybrida*) *PhDHS* gene was isolated, and the role of PhDHS in plant growth was analyzed. PhDHS protein was localized to the nucleus and cytoplasm. Virus-mediated *PhDHS* silencing caused a sectored chlorotic leaf phenotype. Chlorophyll levels and photosystem II activity were reduced, and chloroplast development was abnormal in *PhDHS*-silenced leaves. In addition, *PhDHS* silencing resulted in extended leaf longevity and thick leaves. A proteome assay revealed that 308 proteins are upregulated and 266 proteins are downregulated in *PhDHS*-silenced plants compared with control, among the latter, 21 proteins of photosystem I and photosystem II and 12 thylakoid (thylakoid lumen and thylakoid membrane) proteins. In addition, the mRNA level of *PheIF5A-1* significantly decreased in *PhDHS*-silenced plants, while that of another three *PheIF5As* were not significantly affected in *PhDHS*-silenced plants. Thus, silencing of PhDHS affects photosynthesis presumably as an indirect effect due to reduced expression of *PheIF5A-1* in petunia.

**Significance:** *PhDHS*-silenced plants develop yellow leaves and exhibit a reduced level of photosynthetic pigment in mesophyll cells. In addition, arrested development of chloroplasts is observed in the yellow leaves.

## Introduction

Deoxyhypusine synthase (DHS) is an enzyme thought to be present in all eukaryotic cells (Jenkins et al., 2001). The eukaryotic translation initiation factor eIF5A is identified as the only hypusine-containing protein in eukaryotes (Gordon et al., 1987; Bartig et al., 1992; Kyrpides and Woese, 1998; Park et al., 2010). In animals and yeast, hypusine is formed via a posttranslational modification that involves two enzymes, DHS and deoxyhypusine hydroxylase (DOHH), which catalyzes the first and second steps in the formation of hypusine (Abbruzzese et al., 1986; Park et al., 2006). It has been proposed that plant DHS, similar to its mammalian and yeast counterparts, mediates the first of two reactions required for the post-translational activation of eIF-5A, specifically the formation of deoxyhypusine on the inactive eIF-5A protein (Wang et al., 2003). In plants, recombinant tomato (*Solanum lycopersicum*) and tobacco (*Nicotiana tabacum*) DHS are capable of catalyzing the formation of a deoxyhypusine residue in eIF-5A substrates, respectively (Ober and Hartmann, 1999; Wang et al., 2001).

In most eukaryotes, *DHS* is a single copy gene (Park et al., 2010). A haploid *Saccharomyces cerevisiae* strain with a disruption in the DHS gene was not viable (Sasaki et al., 1996; Park et al., 1998). Similarly, eIF5A depletion led to the growth arrest of enlarged cells in *S. cerevisiae* (Kang et al., 2007). In plants, suppression of DHS led to pleiotropic effects. Detached leaves from DHS-suppressed plants exhibited delayed post-harvest senescence in *Arabidopsis thaliana* (Wang et al., 2003). In *A. thaliana*, leaf-specific suppression of DHS dramatically increased growth and delayed leaf senescence without negative pleiotropic effects (Duguay et al., 2007). In tomato, *DHS* silencing delayed fruit softening and resulted in male sterility plants (Wang et al., 2005a). In canola (*Brassica napus*), transgenic plants with DHS suppression exhibited delayed natural leaf senescence, increased leaf size, and subsequent increases in seed yield and tolerance to chronic sublethal stress (Wang et al., 2005a). These studies provided evidence for senescence-induced DHS in tomato and *A*. *thaliana* tissues that may facilitate the translation of mRNA species required for programmed cell death (Wang et al., 2001, 2003, 2005a). Recent research shows that alterations in the biosynthesis of hypusine promoted by the *DHS* silencing in *A*. *thaliana*, result in a wide variety of phenotypes affecting many biological processes related with development such as control of flowering time, the shoot and root architecture and root hair phenotypes. Additionally this pathway is needed for the adaptation to challenging growth conditions (presence of salt, glucose and the plant hormone ABA in the growth medium) (Belda-Palazón et al., 2016). In addition, recombinant tomato and tobacco DHS are capable of catalyzing the formation of a deoxyhypusine residue in *A*. *thaliana* and tobacco eIF-5A substrates, respectively (Ober and Hartmann, 1999; Wang et al., 2001).

In yeast the key molecular function of eIF5A during translation has been elucidated and it is expected to be fully conserved in every eukaryotic cell (Zuk and Jacobson, 1998; Zanelli and Valentini, 2005; Gregio et al., 2009). eIF5A has been postulated as an RNA-binding protein involved in mRNA transport and metabolism (Xu and Chen, 2001; Xu et al., 2004; Li et al., 2010; Maier et al., 2010). Recent studies have uncovered the crucial function of eIF5A and EF-P, a prokaryotic structural homolog, within the ribosome as a sequence-specific translation factor required for translation of polyproline-rich proteins that may cause ribosome stalling (Doerfel et al., 2013; Gutierrez et al., 2013). In plant, the genetic approaches with transformation for either eIF5A overexpression or antisense have revealed some activities related to the control of cell death processes (Feng et al., 2007; Hopkins et al., 2008; Ren et al., 2013).

In higher plants, the normal development of chloroplasts is associated with leaf color variation (Lv et al., 2015). Chloroplast function is dependent upon proteins encoded both within the plastid and nuclear genomes (Næsted et al., 2004). Many nuclear genes affect overall chloroplast function and encode proteins involved in photosynthesis, plastid metabolism, chloroplast and host cell biogenesis or nuclear-chloroplast traffic and signaling (Barkan et al., 1995; Belcher et al., 2015). Disruptions in these genes lead to abnormal chloroplast development and the sectored chlorotic leaf phenotype. For example, *IMMUTANS (IM)* codes for the plastid terminal oxidase (PTOX) and is involved in regulation of the plastoquinone pool and carotenoid biosynthesis (Wetzel et al., 1994; Wu et al., 1999; Aluru and Rodermel, 2004). The filamentation temperature-sensitive H (FtsH) protease composed of type A (FtsH1 and FtsH5/VAR1) and B (FtsH2/VAR2 and FtsH8) subunits plays an important role in degradation of the photodamaged D1 protein in the PSII repair cycle and probably acts as a molecular chaperone in chloroplasts (Bailey et al., 2002; Sakamoto et al., 2003). Both *ftsh2/var2* and *im* mutants display more severe leaf variegation in high light compared with low light (Rosso et al., 2009). Toc159 protein disruption results in a non-photosynthetic albino phenotype in *A. thaliana* mutant *ppi2* (Agne et al., 2010). Defects in the *vesicle-inducing protein in plastids 1* (*VIPP1*) gene lead to a pale green phenotype in *A. thaliana hcf155* mutant at the early developmental stage (Vothknecht et al., 2012; Zhang et al., 2012). Defects in the *thylakoid formation 1* (*Thf1*) gene cause impaired thylakoid formation and variegated leaves in *A. thaliana* (Wang et al., 2004). In *cpSRP43* (*Chloroplast Signal Recognition Particle 43*) rice (*Oryza sativa*) mutants, chlorophyll and carotenoid levels are significantly reduced, and chloroplast development is impaired (Lv et al., 2015).

Here, full-length petunia *PhDHS* cDNA was cloned, and PhDHS protein was localized in the cytoplasm and nucleus. VIGS-mediated suppression of PhDHS reduced chlorophyll levels and resulted in abnormal chloroplast ultrastructure in leaves, suggesting the crucial function of PhDHS in chloroplast development. Proteome analysis revealed that the proteins involved in photosystem I (PSI) and photosystem II (PSII) were significantly reduced in *PhDHS*-silenced plants, indicating that PhDHS is associated with photosynthesis in petunia.

## Materials and Methods

### Plant Material

Petunia ‘Ultra’ plants purchased from Guangzhou Sanli Horticulture Co., Ltd. were grown under greenhouse conditions (22 ± 2°C, 14-h light/10-h dark). When the plants were ∼10 cm in height, roots, stems, and leaves were collected at the vegetative stage. At anthesis stages, 7–10 petunia flowers were harvested and placed in distilled water. Corollas were collected 1 day after flower opening. Petunia flowers were treated with ethylene according to previously described protocols (Tan et al., 2014). All tissues were frozen in liquid nitrogen and stored at −80°C until use in further experiments. All experiments were conducted at least thrice.

### RNA Extraction, RT-PCR and Cloning of *PhDHS* and *PhCH42* Genes

Total RNA was extracted and reverse transcription PCR (RT-PCR) was performed according to the previously described protocols (Liu et al., 2010). To clone the petunia *PhDHS* and *PhCH42* cDNA by RT-PCR, degenerate primers were designed based on conserved sequences in DHS and CH42 from *A. thaliana* (AtDHS and AtCH42) and tomato (SlDHS and SlCH42) (Supplementary Table S1). The reaction produced 630- and 720-bp PCR products from petunia cDNA. The remaining 5′ and 3′ cDNA sequences were isolated by rapid-amplification of cDNA ends (RACE), and full-length cDNAs for these genes were isolated using the specific primers (Supplementary Table S1).

### Sequence Analysis

The neighbour-joining trees at an amino acid level were generated using DNAMAN software (Lynnon Corporation, Quebec, Canada). The reliability of each branch of the tree was assessed using 1,000 bootstrap replications. An identity search for nucleotides and amino acids was performed using the National Center for Biotechnology Information (NCBI) BLAST network server^1^.

### Quantitative Real-Time PCR Assays

PCR analysis was performed using cDNA extracted from different samples as a template. Specific primers were designed using the sequences of *PhDHS* (Peaxi162Scf00178g01719.1^2^), *PhPDS* (Accession no. AY593974), *PhCH42*, and four *PheIF5A* homologs, *PheIF5A-1* (Peaxi162Scf00274g00004.1), *PheIF5A-2* (Peaxi162Scf00062g00224.1), *PheIF5A-3* (Peaxi162Scf00401g00830.1), and *PheIF5A-4* (Peaxi162Scf00097g00108.1). Quantitative real-time PCR (qPCR) was performed on a LightCycler^®^ 480 Real-Time PCR system (Roche). Samples were subjected to thermal-cycling conditions of DNA polymerase activation at 95°C for 4 min; 40 cycles of 45 s at 95°C, 45 s at 52°C or 55°C, 45 s at 72°C, and 45 s at 80°C; and a final elongation step of 7 min at 72°C. The amplicon was analyzed by electrophoresis and sequenced once for identity confirmation. Primer specificity was determined by melting curve analysis; a single, sharp peak in the melting curve ensured that a single, specific DNA species had been amplified. Quantification was based on analysis of the threshold cycle (Ct) value as described by Pfaffl (2001). Petunia *Actin*, which served as an internal reference gene (accession no. FN014209), was subject to quantitative PCR to quantify cDNA abundance (Supplementary Table S2).

### Subcellular Localization

For protein subcellular localization, the ORF of *PhDHS* was inserted into the modified pSAT-1403TZ (Tzfira et al., 2005) plasmid (which contained GFP under the control of the CaMV 35S promoter) to form the pSAT-35S:GFP-DHS vector. The sequences of all the primers that were used for subcellular localization are described in Supplementary Table S3. The fusion vector and the control vector (35S:GFP) were separately transformed into petunia protoplasts. Rosette leaves of 5- to 6-week-old petunia plants were used for the isolation of protoplasts. Transformation was performed as described by Spitzer-Rimon et al. (2012). The relevant vectors were used in the polyethylene glycol-mediated transformation of the petunia protoplasts. The protoplasts were assayed for fluorescence 12–24 h after transformation. Digital images were captured using a confocal laser scanning system (Nikon ECLIPSE TE2000-E, Nikon Corporation, Tokyo, Japan).

### Construction of VIGS Vectors

Specific forward and reverse primers of *PhDHS*, *PhPDS*, and *PhCH42* were designed to clone approximately 250 bp of the 3′ and 5′ untranslated regions of these genes, and the PCR products were inserted into the pTRV2 vector to form pTRV2-PhDHS, pTRV2-PhDHS-5UTR, pTRV2-PhPDS and pTRV2-PhCH42 vectors, respectively (Supplementary Table S4). *Agrobacterium tumefaciens* (strain GV3101) was transformed with the pTRV2 and pTRV1 vectors, and the derivatives were prepared according to previously described protocols (Spitzer-Rimon et al., 2010). The *Agrobacterium* culture was grown overnight at 28°C in Luria-Bertani medium with 50 mg L^−1^ kanamycin and 200 mM acetosyringone. The cells were harvested and resuspended in inoculation buffer containing 10 mM MES, pH 5.5, 200 mM acetosyringone, and 10 mM MgCl_2_ to an OD_600_ of 10. Following an additional 3-h incubation at 28°C, bacteria containing pTRV1 were mixed with bacteria containing the pTRV2 derivatives in a 1:1 ratio. Next, 200 to 400 mL of this mixture was applied to the cut surface of 4-week-old petunia plantlets after the removal of the apical meristems.

### Pigment Profiling

Four to five leaves were harvested and freeze-dried, and three replicate methanol extractions were prepared for each type of plant using the method of Wellburn (1994). The absorbance of the solution was red at 646.8, 663.2, and 470.0 nm against the solvent (acetone) blank. The individual concentrations of chlorophyll a, chlorophyll b, total chlorophyll, and total carotenoids (xanthophylls and carotenes) were measured by spectrophotometer and calculated using the equations given here (Lichtenthaler, 1987).

$$\mathrm{Chlorophyll a: Ca}=12.25A_{663.2}-2.79A_{646.8}(\mathrm{\mu g per ml solution}).$$

$$\mathrm{Chlorophyll b: Cb}=21.50A_{646.8}-5.10A_{663.2}(\mathrm{\mu g per ml solution}).$$

$$\text{Totalcarotenoids }:\text{ Cx}+\text{c}=(1000{\text{A}}_{470}-1.82{\text{C}}_{\text{a}}-85.02{\text{C}}_{\text{b}})/198(\mathrm{\mu g per ml solution}).$$

### Chlorophyll Fluorescence Measurement

Chlorophyll fluorescence was measured using Dual-PAM-100/F (Walz, Effeltrich, Germany). The third fully expanded leaves from the top were dark-adapted for 30 min prior to measurement. Minimal fluorescence (F0) was measured under a weak pulse of modulating light, and maximal fluorescence (*F*_m_) was induced by a saturating pulse of light (20,000 μmol m^−2^ s^−1^) applied. The values were used to calculate maximum quantum yield of photosystem II (PSII) photochemistry [*F*_v_/*F*_m_ = (*F*_m_ − *F*_0_)/*F*_m_].

### Microscopic Examination

Confocal laser microscopic, transmission electron microscopic and optical microscopic examinations were performed according to previous studies (Waters et al., 2008; Yang et al., 2017).

### Iodine and Trypan Blue Staining and Measurement of Ion Leakage

Starch was visualized in petunia rosettes by iodine staining as previously described (Bahaji et al., 2011; Ovecka et al., 2012), and trypan blue was used to reveal dead cells in petunia leaves using optical microscopy (Koch and Slusarenko, 1990).

Membrane ion leakage was determined by measuring electrolytes released from the leaves according to previous protocal (Song et al., 2016). Leaf samples were immersed into deionised water at 25°C with gentle shaking for 30 min. The conductivity was measured using a conductivity meter (CON 510, Oakton, VA, United States).

### Paraffin Sections

Paraffin sections were prepared according to our previous studies (Yang et al., 2017). Briefly, the leaves from plants were cut into 5 mm × 5 mm × 5 mm pieces, fixed in FAA fixative solution (every 100 ml of FAA fixative solution contains 90 ml of 50% or 70% ethanol, 5 ml of acetic acid, and of 5 ml formalin) for 24 h at 25 ± 2°C, and then washed in running water for 24 h. Samples were stained with haematoxylin for 4 days, washed for another 24 h, and dehydrated in increasing grades of ethanol. A graded chloroform series was used for clearing, and the samples were embedded in paraffin. Paraffin sections were cut to a thickness of 8 μm on a Leica RM2235 followed by dewaxing with xylene. Finally, slides were sealed with neutral resin. Sections were observed and photographed with a Zeiss Scope.A1 microscope, and the thickness of the leaves was calculated according to the scale of the microscope.

### Protein Extraction, Trypsin Digestion, TMT Labeling, HPLC Fractionation and LC-MS/MS Analysis

Protein extraction, trypsin digestion, TMT labeling and HPLC fractionation were performed according to our previous study (Guo et al., 2016). LC-MS/MS analysis was performed according to previously described protocols (Wu et al., 2015). Three biological replicates were performed.

### Database Search

The resulting MS/MS data were processed with MaxQuant (v.1.4.1.2) (Cox and Mann, 2008). Tandem mass spectra were searched against a database (40,341 sequences) created based on the RNA sequencing of petunias in our previous study (Guo et al., 2016).

### Bioinformatics Analysis

Bioinformatics analysis was performed according to previously described protocols (Xie et al., 2015). Gene Ontology (GO) term association and enrichment analysis were performed using DAVID (Database for Annotation, Visualization and Integrated Discovery^3^). The KEGG pathway database was used to identify enriched metabolic pathways^4^.

### Protein Quantitative Ratio Analysis

Protein quantitative ratio was calculated as the median ratio of all unique peptides. Student’s *t*-test was performed to investigate the effects of differentially expressed proteins (DEPs). To meet the conditions for Student’s *t*-test, logarithmic transformation was performed to obtain the ratio of all peptides. Then, Student’s *t*-test was employed to calculate the *p*-value.

### Western Blotting

Western blot analyses were performed according to the methods of Guo et al. (2017). The synthetic peptides (KRWLHFFMLFVPVT, PKIQGNLSSDEE-KYS and RIPVFCPGLTDGSLG) of three proteins, PsbD (Peaxi162Scf00060g00176.1), PsbQ1 (Peaxi162Scf00222g00618.1) and PhDHS (Peaxi162Scf00178g01719.1), were used as an antigen for antibody production in rabbits ^5^ (PTM BioLab, Inc.), and these antibodies were used for blotting analysis. Proteins were separated using SDS-polyacrylamide gel electrophoresis (PAGE; 10% acrylamide gels) and blotted onto nitrocellulose membranes. The membrane was blocked with 5% skim milk and 0.05% Tween 20 in Tris-buffered saline (50 mM Tris-HCl, pH 8.0, 150 mM NaCl). Purified antibodies were used at a concentration of 50 mg/ml. The membrane was washed with 0.05% Tween 20 in Tris-buffered saline and then reacted with horseradish peroxidase-conjugated goat anti-rabbit IgG (Pierce) at a dilution of 1:20,000. Detection was achieved using SuperSignal West Femto^TM^ (Pierce). Three biological replicates were performed.

## Results

### Isolation and Sequence Analyses of PhDHS cDNAs

Full-length *DHS* cDNA was isolated from petunia ‘Ultra’ and was designated as *PhDHS*, which was predicted to encode a 375-amino acid protein. Multiple sequence alignments of DHS of petunia, *A. thaliana*, *S. cerevisiae*, *Drosophila melanogaster*, and *Homo sapiens* are presented in Supplementary Figure S1A. The phylogenetic trees based on evolutionary distances were constructed from DHS amino acid sequences using the DNAMAN program (Supplementary Figure S1B).

The deduced amino acid sequence of PhDHS shares 74.2, 57.0, 54.7, and 59.7% identity with *A. thaliana* AtDHS, *S. cerevisiae* ScDHS, *D. melanogaster* DmDHS, and *H. sapiens* HsDHS, respectively (Supplementary Table S5). DHS protein alignment performed with the DNAMAN program is presented in Supplementary Figure S1A. These results revealed considerable conservation of sequence identity, especially in the C-terminal active site, which includes a conserved region of six amino acids from Glu 330 to Lys 335 (Supplementary Figure S1A) (Yan et al., 1996; Duguay et al., 2007). The NAD-binding site (positions 97–349) is strongly conserved in all species.

### Expression Profile of *PhDHS* Gene

**FIGURE 1:**
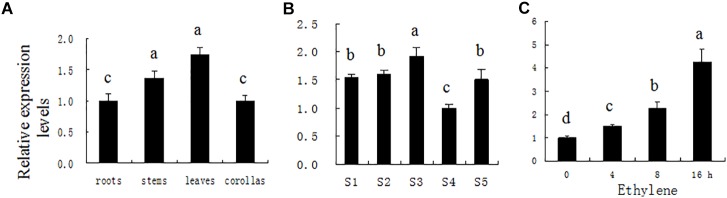
*PhDHS* expression patterns determined by quantitative real-time PCR in different organs **(A)** during leaf development **(B)** and in leaf in response to 2 μl l^−1^ of exogenous ethylene **(C)**. Petunia *Actin* (accession no. FN014209) served as an internal reference gene. S1, young leaves at 0.5 cm; S2, young leaves at 1.0 cm; S3, young leaves at 2.5 cm; S4, mature leaves; S5, aging leaves. The relative expression levels are presented as fold-change values. The data are presented as the mean ± SD (*n* = 3). Different letters indicate significant differences at the *P* = 0.05 level.

*PhDHS* expression was examined in different plant organs at different leaf developmental stages and in response to ethylene, a flower-senescence hormone by quantitative real-time PCR (qPCR), given that a previous study suggested that *DHS* is involved in fruit softening and senescence (Wang et al., 2005a). *PhDHS* transcription was strong in the leaves, and no significant difference was noted between roots and corollas (Figure 1A).

To assess *PhDHS* expression during leaf development, leaf development is divided into five stages: S1 (young leaf, 0.5 cm in length), S2 (young leaf, 1.0 cm in length), S3 (young leaf, 2.5 cm in length), S4 (mature leaf, approximately 4.5 cm), and S5 (aging leaf). High *PhDHS* expression is noted in young and senescence stages, whereas low expression is observed in the mature stage (Figure 1B). In addition, 2 μL L^−1^ ethylene treatment significantly increased *PhDHS* expression in leaves (Figure 1C).

### PhDHS Protein Is Localized to the Cytoplasm and Nucleus

**FIGURE 2:**
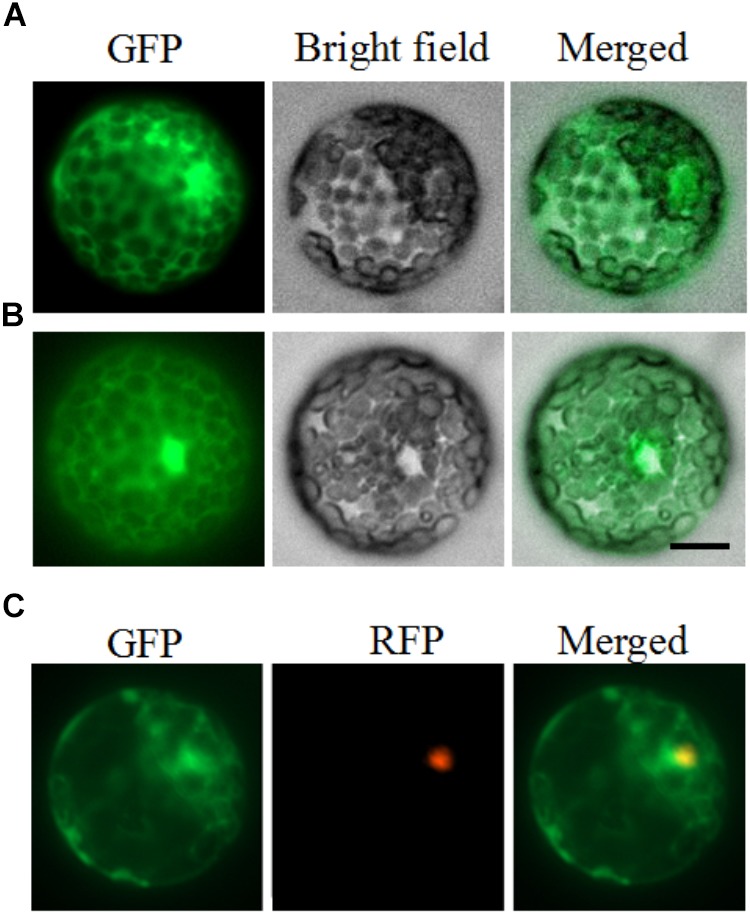
Subcellular localization of PhDHS. Petunia protoplasts were transfected with a construct carrying GFP or PhDHS-GFP under the control of CaMV 35S promoter to assess subcellular localization. **(A)** Expression of PhDHS-GFP fusion protein. **(B)** Expression of GFP protein. **(C)** Subcellular localization of PhDHS-GFP with EOBI-RFP as makers. All images were captured with a confocal laser scanning system. Scale bar: 5 μm.

To examine the cellular localization of PhDHS in plant cells, the full-length form of the protein fused to GFP at the C-terminal end was constructed and transiently expressed in petunia leaf protoplasts under the control of the CaMV 35S promoter. After 16–24 h, accumulation of the PhDHS-GFP fusion protein was detected in the cytoplasm and nucleus of petunia protoplasts (Figure 2A,B). To further verify the fluorescence signal of PhDHS-GFP fusion protein in the nucleus, we use EOBII-RFP (red fluorescent protein) as a nuclear marker given that it was previously demonstrated that EOBI are nuclear-localized MYB factors (Spitzer-Rimon et al., 2010, 2012; Liu et al., 2017). Localization of the PhDHS-GFP fusion protein was determined by visualization with a fluorescence microscope. The results showed that the fluorescence signal of the PhDHS-GFP fusion protein appears in the cytoplasm and nucleus (Figure 2C).

### Phenotypes of *PhDHS*-Silenced Plants

To suppress *PhDHS* expression, a VIGS system based on the pTRV2 vector was used in petunia ‘Ultra’ (Tan et al., 2014). An approximately 250-bp fragment of the 3′ untranslated sequence of *PhDHS* cDNA was inserted into the pTRV2 vector to form the pTRV2-PhDHS vector. Thirty to thirty-five petunia plants were used for infection. PhDHS expression in both mRNA and protein level in leaves of pTRV2-PhDHS-infected plants decreased significantly compared with pTRV2-infected plants (Supplementary Figure S2).

**FIGURE 3:**
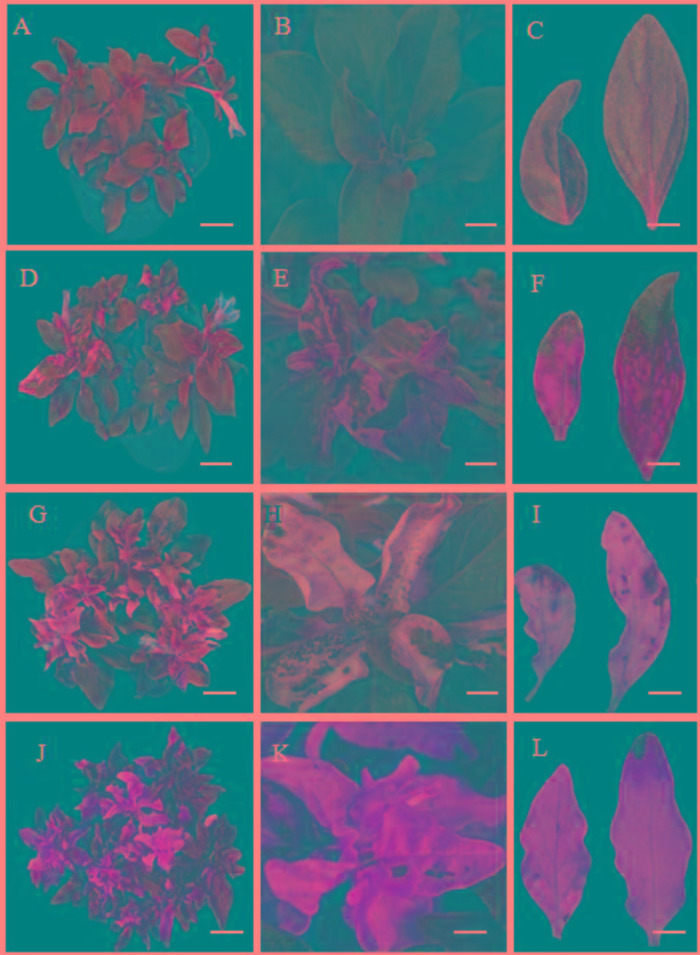
Phenotype of pTRV2 empty vector-infected plants (control) and *PhDHS*-, *PhPDS-*, and *PhCH42*-silenced plants. Seven-week-old plants exhibiting different leaf color in pTRV2 control **(A–C)**, *PhDHS*- **(D–F)**, *PhPDS*- **(G–I)**, and *PhCH42* **(J–L)**-silenced plants. **(A,D,G,J)** Scale bar, 6.0 cm. **(B,E,H,K)** Scale bar, 1.3 cm. **(C,F,I,L)** Scale bar, 1.0 cm.

Four to five weeks after petunia seedlings were infected with a mixture of *A. tumefaciens* transformed with the pTRV2-PhDHS vector and *A. tumefaciens* was transformed with pTRV1 according to the protocol of Liu et al. (2002), the newly emerged leaves of pTRV2 empty vector-infected plants (control) remained green (Figure 3A–C), whereas those of pTRV2-PhDHS-infected plants exhibited a chlorotic leaf phenotype (Figure 3D–F). Some leaves were completely yellow. Some leaves exhibited reduced chlorophyll in the vicinity of vascular bundles of the systemic leaves. Some plants had leaves with yellow sectors (Figure 3D–F). Additionally, sepals also appeared yellow in *PhDHS*-silenced plants (Supplementary Figure S3).

As a control, we silenced the petunia *Phytoene desaturase* (*PhPDS*) gene (Accession no. AY593974), which results in a photobleaching phenotype due to inhibition of carotenoid biosynthesis (Ratcliff et al., 2001; Liu et al., 2002; Turnage et al., 2002), and the homolog of the *A. tumefaciens Chlorata42* gene Petunia *Chlorata42* (*PhCH42*, Peaxi162Scf00269g00514.1), which produces a yellow-leaf phenotype (Ratcliff et al., 2001; Liu et al., 2002; Turnage et al., 2002). *PDS* and *CH42* have been used as marker genes for the effectiveness of VIGS in several studies (Burch-Smith et al., 2006).

The sectored chlorotic leaves and sepals were obvious in *PhPDS*- and *PhCH42*-silenced plants. The silencing of *PhPDS* and *PhCH42* produces typical white and yellow colors, respectively, in leaves, which occurs in the absence of the genes products (Figure 3G–L). Of note, *PhCH42*-silenced plant exhibit more yellow leaves compared with *PhDHS*-silenced plants (Figure 3J–L). No visible symptoms of pTRV2 infection were observed in these plants, and these plants were indistinguishable from pTRV2-infected plants. The levels of gene silencing were monitored by qPCR, and an 83 and 85% reduction in *PhPDS* and *PhCH42* transcriptional levels, respectively, was noted in sectored chlorotic leaves (Supplementary Figures S4A, B).

**FIGURE 4:**
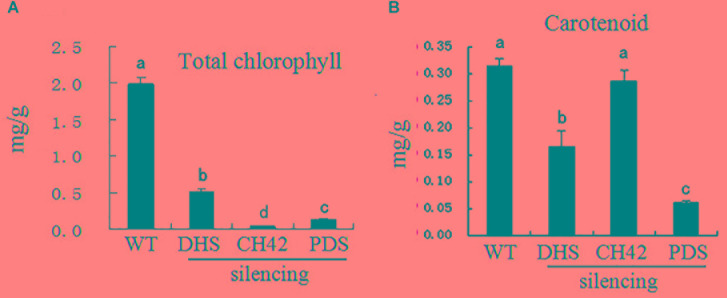
Effects of *PhDHS*, *PhPDS* and *PhCH42* silencing on the photosynthetic pigment content of leaf mesophyll cells from 5-week-old plants. **(A)** Total chlorophyll; **(B)** Carotenoid. Data are presented as the mean ± SD (*n* = 3). Different letters indicate significant differences at the *P* = 0.05 level.

Leaf color is typically associated with chlorophyll and carotenoid levels (Kim and An, 2013). We thus investigated chlorophyll and carotenoid levels in leaves from *PhDHS*-, *PhPDS-*, and *PhCH42*-silenced plants. As shown in Figure 4, the silencing of three genes significantly reduced chlorophyll content in leaves compared with the control. Leaves from *PhPDS*- and *PhCH42*-silenced plants exhibited reduced chlorophyll content compared with *PhDHS*-silenced plants. Both *PhDHS* and *PhPDS* silencing reduced carotenoid content in leaves compared with the control, whereas *PhCH42* silencing produced the same carotenoid level as the control. In addition, leaves from *PhPDS*-silenced plants exhibited less carotenoid levels compared with *PhDHS*-silenced plants.

Effects of *PhDHS* silencing on maximum quantum yield of PSII photochemistry (*F*_v_/*F*_m_) in petunia leaves were examined. Compared to the control, there was a significant decrease in *F*_v_/*F*_m_ in leaves of *PhDHS* silencing and the values of *F*_v_/*F*_m_ were decreased by 18.55% (Supplementary Figure S5), which showed PSII activity in the leaves of *PhDHS* silencing were reduced.

To visualize starch accumulation, we used iodine staining of young leaves at the S3 stage and mature leaves from 5-week-old *PhDHS*-silenced and pTRV2-infected plants. The staining revealed that the control leaves retained more starch compared with *PhDHS*-silenced plants (Supplementary Figure S6).

In addition, *PhDHS* silencing resulted in extended leaf longevity. The detached leaves exhibited a normal configuration in *PhDHS*-silenced plants, whereas leaf senescence was noted 10 days after harvest in pTRV2-infected plants (Supplementary Figure S7A). Trypan blue staining of leaves revealed an increased number of dead cells in pTRV2-infected plants compared with *PhDHS*-silenced leaves 10 days after harvest (Supplementary Figure S7B). Moreover, measurement of ion leakage, which indicates the plasma membrane integrity, revealed that the plasma membrane of leaves of pTRV2-PhDHS-infected plants was more intact than leaves of pTRV2-infected plants 5 weeks after infection, which further confirmed that *PhDHS* silencing delayed senescence (Supplementary Figure S8).

The cross-sections of leaves pTRV2- and pTRV2-PhDHS-infected plants were prepared, revealing that mature leaves of pTRV2-PhDHS-infected plant were thicker compared with control (Supplementary Figure S9). The thickness of leaves in *PhDHS*-silenced and pTRV2-infected plants was 83 ± 6 μm and 63 ± 7 μm, respectively. In addition, the height of pTRV2-PhDHS-infected plants (22.76 ± 0.90 cm) did not show significantly difference compared with that of the pTRV2-PhDHS-infected plants (22.13 ± 1.84 cm).

Although the proper gene silencing for *PhDHS* was showed, the phenotypes uncovered and assigned to the expression level of *PhDHS* cannot be allocated exclusively to down-regulation of *PhDHS* gene expression as off-targets can be generated by the approach used. So, a 253-bp fragment of the 5′ untranslated sequence of *PhDHS* cDNA was inserted into the pTRV2 vector to form the pTRV2-PhDHS-5UTR vector. Four to five weeks after infection, a phenotype similar to the plants treated with pTRV2-PhDHS was exhibited in the petunia plants treated with pTRV2-PhDHS-5UTR (Supplementary Figure S2). The newly emerged leaves of pTRV2-PhDHS-5UTR-infected plants also exhibited a chlorotic leaf phenotype (Supplementary Figure S10). *PhDHS* expression in leaves of pTRV2-PhDHS-5UTR-infected plants decreased significantly compared with pTRV2-infected plants (Supplementary Figure S11).

### PhDHS Suppression Affects Chloroplast Development

**FIGURE 5:**
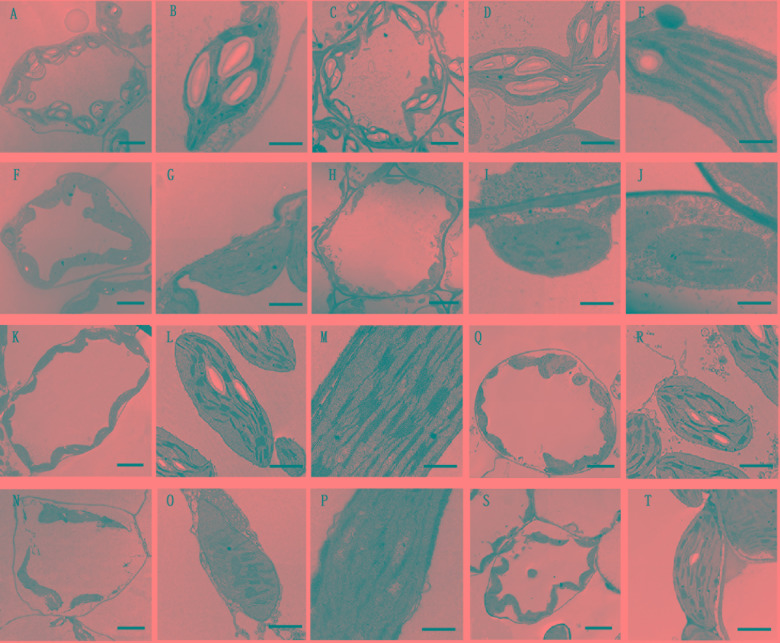
Effects of *PhDHS* silencing on chloroplast development. **(A–J)** Chloroplast ultrastructure of mesophyll chloroplasts in the S1 **(A,B,F,G)** and S2 **(C–E,H–J)** stages of leaves in 5-week-old pTRV2 empty vector-infected **(A–E)** and *PhDHS*-silenced **(F–J)** plants. **(A,C,F,H)** Overviews depicting the chloroplast morphology and arrangement within the cells (scale bar, 5 μm). **(B,D,G,I)** Inside chloroplast views depicting thylakoid arrangement (scale bar, 1 μm). **(E,I)** Higher magnification depicting the grana stacking (scale bar, 0.5 μm). **(K–P)** Chloroplast ultrastructure of mesophyll chloroplasts in the S3 stage of leaves in 5-week-old pTRV2 empty vector-infected **(K–M)** and *PhDHS*-silenced **(N–P)** plants. **(K,N)** Overviews depicting the chloroplast morphology and arrangement within the cells (scale bar, 5 μm). **(L,O)** Inside chloroplast views depicting thylakoid arrangement (scale bar, 1 μm). **(M,P)** Higher magnification depicting the grana stacking (scale bar, 0.5 μm). **(Q–T)** Chloroplast ultrastructure in the leaves of S4 stage in pTRV2 empty vector-infected **(Q,R)** and *PhDHS*-silenced **(S,T)** plants. **(Q,S)** Overviews depicting the chloroplast morphology and arrangement within the cells (scale bar, 5 μm). **(R,T)** Inside chloroplast views depicting thylakoid arrangement (scale bar, 1 μm).

Given that the chlorophyll content deceased in *PhDHS*-silenced plants, we hypothesized that the *PhDHS* silencing phenotype resulted from the abnormal development of chloroplasts in mesophyll cells. To test this hypothesis, we observed the ultrastructure of chloroplasts of *PhDHS*-silenced and pTRV2-infected plants at four developmental stages, S1, S2, S3 and S4, by transmission electron microscopy (Figure 5).

In the S1 and S2 stages of leaves, the thylakoid arrangement in chloroplasts of middle parts of leaves in *PhDHS*-silenced and pTRV2-infected plants was regular (Figure 5A–J). However, the size of grana in chloroplasts and starch granules in chloroplasts in *PhDHS*-silenced plants were reduced compared with the control.

In S3 stage leaves from pTRV2-infected plants, individual ellipsoidal chloroplasts of the middle leaves were aligned along the cytoplasmic membrane. These chloroplasts contained starch granules, and their internal structures were completely developed (Figure 5K–M). The lamellae in chloroplasts were oriented along the convex side facing the interior of the cell (Figure 5K). In contrast, the chloroplasts of yellow areas of *PhDHS*-silenced leaves were detached from the plasma membrane (Figure 5N). Grana thylakoids were significantly decreased (or even completely lost) with discontinuous distribution in *PhDHS*-silenced plants. A loose arrangement of grana was noted, and some grana were even arranged in the vertical axis of the chloroplast in *PhDHS*-silenced plants (Figure 5O,P). Fewer starch granules in chloroplasts were observed in *PhDHS*-silenced plants (Figure 5N,P).

In S4 stage leaves, grana thylakoids and starch granules in some chloroplasts were slightly increased compared with S3 stage leaves in *PhDHS*-silenced plants; however, the levels were slightly reduced compared with the control (Figure 5Q–T).

### Effects of *PhDHS* Silencing on the mRNA Levels of *PheIF5As*

**FIGURE 6:**
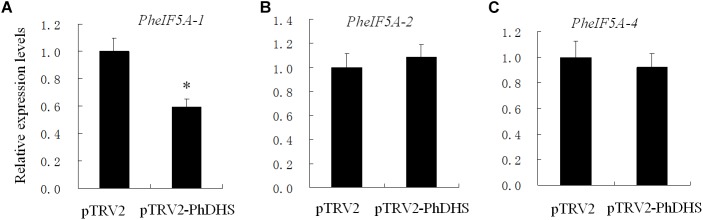
Effects of *PhDHS* silencing on the expression of *PheIF5A-1* **(A)**, *PheIF5A-2* **(B)**, and *PheIF5A-4* **(C)** by quantitative real-time PCR. Petunia *Actin* (accession no. FN014209) served as an internal reference gene. Relative expression levels are presented as fold-change values (1 = time 0). Data are presented as the means ± SD (*n* = 3).

Given that previous studies suggested that eIF5A is the only substrate of DHS, the effect of *PhDHS* silencing on PheIF5A expression was examined. Four (PheIF5A-1, PheIF5A-2, PheIF5A-3, and PheIF5A-4) and three (AteIF5A-1, At1g13950; AteIF5A-2, At1g26630; AteIF5A-3, and At1g69410) eIF5A homologs are present in the petunia and *A. thaliana* genome, respectively (Hopkins et al., 2008). The phylogenetic tree demonstrated that the predicted four PheIF5A proteins were classified as one group, whereas three AteIF5A proteins belong to another group (Supplementary Figure S12). The qPCR assays showed that in the leaves of *PhDHS*–silenced plants, *PheIF5A-2* and *PheIF5A-4* mRNA levels did not significantly change compared with levels in pTRV2-infected plants, whereas *PheIF5A-1* expression was significantly reduced (Figure 6). In addition, *PheIF5A-3* mRNA was not detected in leaves of both pTRV2-infected and pTRV2-PhDHS-infected plants. We further examined the expression patterns of *PheIF5A-1, PheIF5A-2*, and *PheIF5A-4* in different organs. The results revealed that *PheIF5A-1* and *PheIF5A-2* have higher expression level in corollas and leaves than that in stems and roots, while *PheIF5A-4* show highest expression level in roots (Supplementary Figure S13).

### PhDHS Suppression Resulted in the Reduction of Proteins Associated With Photosynthesis

To characterize the sectored chlorotic leaves in *PhDHS*-silenced plants, we quantitatively investigated the petunia proteome of S3 stage leaves in pTRV2-infected plants and *PhDHS*-silenced plants by iTRAQ. Tandem mass spectra were searched against the transcriptome sequences (SRA accession: SRP077541) we previously constructed to analyze the proteome (Guo et al., 2016). In the proteome, a total of 3763 proteins were identified, among which 3101 proteins were quantified. The fold-change cut-off established a quantitative ratio greater than 1.5 or less than 0.66 as significant. Among the quantified proteins, 308 proteins were upregulated and 266 proteins were downregulated in *PhDHS*-silenced plants compared with control with high repeatability (Supplementary Figure S14 and Supplementary Data 1).

**FIGURE 7:**
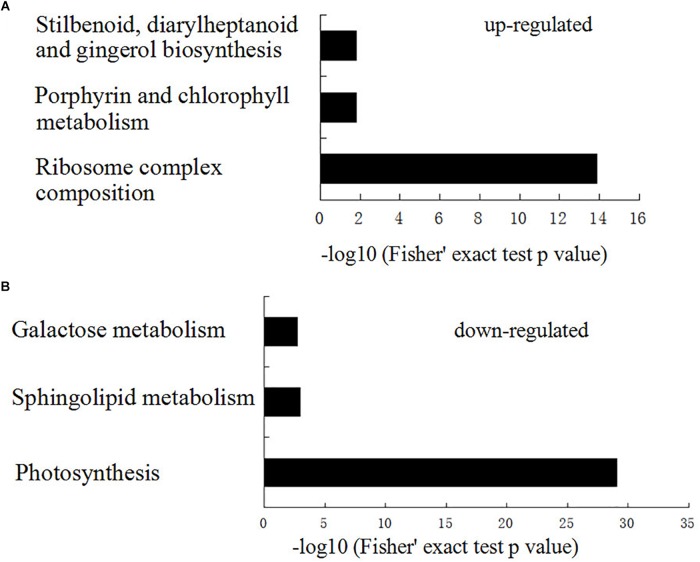
KEGG pathway-based enrichment analysis of up- **(A)** and down- **(B)** regulated proteins in *PhDHS*-silenced plants compared with control.

To elucidate the functional differences of the upregulated and downregulated proteins, the quantified proteins were analyzed for KEGG pathway enrichment based on clustering analysis (Supplementary Data 2). KEGG pathway enrichment-based clustering analysis revealed that ribosome complex composition, porphyrin and chlorophyll metabolism, and stilbenoid, diarylheptanoid, and gingerol biosynthesis were the most prominent pathways enriched in quantiles with increased protein levels in *PhDHS*-silenced leaves (Figure 7). In contrast, photosynthesis, sphingolipid metabolism, and galactose metabolism pathways were reduced in *PhDHS*-silenced leaves (Figure 7 and Supplementary Figures S15–S20).

Given that DHS catalyzes eIF5A function in protein translation, we focused on our attention on the downregulated proteins. Ten proteins among 16 subunit proteins in the PSI complex (PsaC, PsaD1, PsaD2, PsaE1, PsaE2, PsaF, PsaK, PsaL1, PsaL2, and PsaN) and 11 proteins among 27 subunit proteins in the PSII complex (Psb27, PsbB, PsbC, PsbD, PsbO2A, PsbO2B, PsbP2, PsbQ1, PsbQ2A, PsbQ2B, and PsbR) were significantly reduced (Supplementary Table S6). Among them, PsbO2A and PsaE protein levels in *PhDHS*-silenced plants were reduced to one-fifth of those in control. In addition, twelve thylakoid membrane or lumenal proteins were also significantly reduced in *PhDHS*-silenced leaves compared with control (Supplementary Table S7).

### Confirmation of the Petunia Proteome by Western Blotting

To confirm the petunia proteome results of S3 stage leaves in pTRV2-infected plants and *PhDHS*-silenced plants by iTRAQ, we random selected 2 protein, photosystem II D2 protein (PsbD, Peaxi162Scf00060g00176.1) and PsbQ1 (Peaxi162Scf00222g00618.1), to perform western blotting using the antibodies raised against these proteins. The results showed that protein abundance was reduced in *PhDHS*-silenced plants compared with plants treated with pTRV2 vector (Supplementary Figure S21), which is consistent with that in the petunia proteome as demonstrated by iTRAQ.

## Discussion

In the present study, the *PhDHS*-silencing phenotype in petunia was obtained by using the VIGS system, and we demonstrated that *DHS* plays important roles in chloroplast development.

*DHS* and *eIF5A* full-length cDNAs were isolated from *A. thaliana*, canola, tomato, tobacco and rice (Chamot and Kuhlemeier, 1992; Mehta et al., 1994; Wang et al., 2001; Wang et al., 2005b). Studies to date have indicated that *DHS* is a single gene, whereas eIF5A is encoded by a multi-gene family (Wang et al., 2005a; Duguay et al., 2007). In this study, full-length petunia *PhDHS* cDNA was isolated. The amino acid identity between the petunia PhDHS and the *H. sapiens* enzyme is 59.7%. The identity between the petunia PhDHS and the *D. melanogaster* enzyme is 54.7%, the lowest identity among the petunia, *H. sapiens*, the *A. thaliana*, *D. melanogaster*, and *S. cerevisiae* enzyme, suggesting that DHS is highly conserved in eukaryotes.

Similar to the expression of *AtDHS* and *SlDHS* observed in *A. thaliana* and tomato (Wang et al., 2005a), *PhDHS* expression changes both spatially and temporally as development progresses in petunia. *PhDHS* was expressed at high levels in young and senescent leaves (Figure 1B). This finding is consistent with the finding that suppression of DHS resulted in abnormal development of chloroplasts in leaves and delayed leaf senescence (Figure 5 and Supplementary Figure S7). Treatment with the senescence hormone ethylene increased *PhDHS* expression, which further implicated the probable involvement of *PhDHS* in leaf senescence. This finding is consistent with previous studies demonstrating that DHS protein levels in leaves were upregulated in response to both natural senescence and stress-induced premature senescence in tomato and *A. thaliana* (Wang et al., 2001, 2003, 2005a).

Although VIGS-mediated silencing of *PhDHS*, *PhPDS*, and *PhCH42* resulted in sectored chlorotic leaf phenotype, the leaf color in the plants with three genes silenced was not completely identical. Moreover, the carotenoid and chlorophyll content in *PhDHS*-silenced plants differed from that in *PhPDS*- and *PhCH42*-silenced plants. These results indicate that the molecular mechanism of the sectored chlorotic leaves differed among plants with these genes silenced. PhPDS and PhCH42 are the key enzymes of carotenoid and chlorophyll biosynthesis, respectively (Ratcliff et al., 2001; Liu et al., 2002; Turnage et al., 2002).

Measurements of photosynthetic pigments revealed that carotenoid levels in *PhDHS*-silenced plants were 50% of that in pTRV2-infected plants, whereas chlorophyll a and b levels in *PhDHS*-silenced plants were approximately 27 and 20% of those in pTRV2-infected plants, respectively. This finding indicated that loss of *DHS* function has a greater effect on chlorophyll levels than carotenoid levels. Iodine staining of leaves revealed that the chloroplasts of pTRV2-infected leaves retained more starch granules compared with *PhDHS*-silenced plants (Supplementary Figure S6). The reduced starch granules in the chloroplasts of leaves of *PhDHS*-silenced plants might be attributed to the reduction in total chlorophyll levels and abnormal chloroplast development.

Recent research shows that *AtDHS* silencing resulted in early flowering by the increasing of expression of *AtFT* gene, one of key genes involved in the transition process, and alterations of shoot and root architecture (Belda-Palazón et al., 2016). *PhDHS* silencing did not significantly change the height of plants and flowering time and it is probably because the seedlings used to infect by using VIGS method have already bloomed and are tall enough in this study.

Transmission electron microscopy revealed abnormal chloroplast development in *PhDHS*-silenced plants (Figure 5). These results suggested that PhDHS was required for chloroplast development. Moreover, *PhDHS* mRNA levels were high in young leaves when chloroplasts were developing. In addition, the mesophyll chloroplasts of leaves in *PhDHS*-silenced plants exhibited frequent changes in their thylakoid orientation and fewer thylakoids per granal stack (Figure 5).

The proteome assay demonstrated that *PhDHS* silencing resulted in the reduction of proteins involved in photosynthesis pathways, suggesting that *PhDHS* is associated with photosynthesis. PSI and PSII complexes are located in the thylakoid membrane, and the assembly of PSI and PSII complexes is associated with thylakoid formation and chloroplast development (Pogson and Albrecht, 2011). In addition, lipid and protein biosynthetic pathways are involved in the integration and formation of thylakoids (Benning, 2009). In this study, 21 proteins in PSI and PSII complexes and 12 thylakoid membrane or lumenal proteins were significantly reduced in *PhDHS*-silenced leaves compared with control, suggesting that the reduction of photosynthesis proteins could result in sectored chlorotic leaves and abnormal chloroplast development in *PhDHS*-silenced plants. These results further supported the involvement of PhDHS in chloroplast development. *PhDHS* silencing significantly decreased the expression of *PheIF5A-1* and did not significantly change the transcription levels of *PheIF5A-2* and *PheIF5A-4* (Figure 6), while in the proteome of this study, the protein levels of PheIF5A1, PheIF5A2, and PheIF5A4 in the leaves of *PhDHS*-silenced plants increase by 1.224, 1.362, and 1.537 times compared with those in control, while the peptides of PheIF5A3 were not detected, respectively (Supplementary Data 1). These results show that *PhDHS* silencing promotes the translation of *PheIF5A1*, *PheIF5A2*, and *PheIF5A4* mRNA since *PhDHS* silencing did not increase their mRNA levels. The up-regulation of the protein levels of PheIF5A1, PheIF5A2, and PheIF5A4 might be attributed to the decrease of hypusinated PheIF5As resulting from *PhDHS* silencing in petunia and this may indicate that feedback control is involved in the regulation of PheIF5As translation. *PheIF5A-1*, *PheIF5A-2*, *PheIF5A-4*, and *PhDHS* exhibit high mRNA levels in petunia leaves (Figure 1 and Supplementary Figure S12). Similar to PhDHS, AteIF5A-2 is localized to the cytoplasm and nucleus (Hopkins et al., 2008). It is not ruled out that *PheIF5A-1*, *PheIF5A-2*, and *PheIF5A-4*, as possible substrates of PhDHS, might play a role in abnormal chloroplast development resulting from *PhDHS* silencing in petunia.

In addition, it is shown here proteins of PSI and PSII are downregulated in *PhDHS*-silenced plants and most PSI and PSII proteins are encoded in the chloroplast and translated there, while PhDHS is localized to the cytoplasm and nucleus. Presumably the defects in photosynthesis in this study are a secondary effect. It is possible that the down-regulation of *PheIF5A-1* mRNA in *PhDHS*-silenced plants affects the elongation or termination of the protein encoding by the nuclear genes involved in thylakoid formation, which resulted in the reduction of chloroplast encoded proteins.

*PhDHS* silencing resulted in extended leaf longevity and increased thickness in leaves. These effects are consistent with *DHS* silencing in tomato, *A. thaliana* and canola (Duguay et al., 2007). Similarly, suppression of DHS also delays postharvest senescence of cut carnation flowers (Hopkins et al., 2007). In addition, *A. thaliana* eIF5A, specifically AteIF5A-2, is involved in plant growth and development by regulating cell division, cell growth, and cell death and pathogen-induced cell death and development of disease symptoms (Feng et al., 2007; Hopkins et al., 2008; Ren et al., 2013). Thus, hypusination of eIF5A appears to be an important element of the regulation of senescence or cell death.

The sectored chlorotic leaves, which appeared in *PhDHS*-silenced petunias in this study, was not reported in constitutive suppression of DHS in *A. thaliana*, tomato and canola (Wang et al., 2003, 2005a,b; Duguay et al., 2007). In *A. thaliana* and tomato, both chlorophyll content and PSII activity proved to be higher for DHS-suppressed leaves than for corresponding wild-type leaves (Wang et al., 2003, 2005a). In contrast, both chlorophyll content and PSII activity in the leaves of *PhDHS* silencing were reduced compared with the control leaves. These results indicated functional differences of DHSs among different species.

Based on previous studies (Wang et al., 2003; Park et al., 2010), eIF5As are the only substrate of DHS. Although there are four and three eIF5A homologs in petunia and *A. thaliana* genome, respectively, there is only one DHS isoform, implying that the single isoform of DHS is capable of modifying all four isoforms of eIF-5A. Suppression of DHS and reduction of eIF-5A levels in *A. thaliana* and/or tomato has dramatic effects on growth and development (Wang et al., 2003, 2005b). These pleiotropic effects arising from the suppression of DHS in *A. thaliana* and/or tomato indicate that eIF-5A plays a central role in plant growth and development. The functional differences of DHSs between different species might result from different eIF5A substrates or different downstream events of DHS enzyme and possible novel specific functions for eIF5A could explain the phenotypes of *PhDHS* silencing, and further studies are required.

It seems to be contradictory that PhDHS removal results in chlorophyll loss and chloroplast disruption, which reduces photosynthesis and sugar and starch accumulation and should be associated with accelerated senescence of leaves (Oda-Yamamizo et al., 2016) and that *PhDHS* silencing delayed senescence. It is possible that sugar and starch output from leaves is reduced, whereas the retained portion in the leaves is not significantly reduced in *PhDHS*-silenced plants (Wang et al., 2005a). Alternatively, *PhDHS* silencing results in increased thickness in leaves (Supplementary Figure S8) or likely inhibits the expression of senescence-associated genes at transcriptional and translational levels, which adequately compensates for the effects of the reduction in energy.

The proteome assay showed that besides photosynthesis pathways, the metabolism pathways of sphingolipid, which plays an important role in modulating plant programmed cell death associated with defense (Wang et al., 2008), and the metabolism pathways of galactose, which is involved in xyloglucan synthesis in Arabidopsis (Kong et al., 2015), were reduced in *PhDHS*-silenced leaves (Supplementary Figures S15–S20). In addition, ribosome complex composition, porphyrin and chlorophyll metabolism, and stilbenoid, diarylheptanoid and gingerol biosynthesis were the most prominent pathways enriched in quantiles with increased protein levels in *PhDHS*-silenced leaves.

## Accession Numbers

The mass spectrometry proteomics data have been deposited to the ProteomeXchange Consortium (Vizcaíno et al., 2010) via the Proteomics Identification Database partner repository with the dataset identifier PXD012227.

## Author Contributions

YY and JL planned and designed the research. JL, XC, SZ, BD, LP, JM, and QW performed the experiments, conducted fieldwork, analyzed the data etc. YY and JL wrote the manuscript.

## Conflict of Interest Statement

The authors declare that the research was conducted in the absence of any commercial or financial relationships that could be construed as a potential conflict of interest.

## Footnotes

1 https://blast.ncbi.nlm.nih.gov/Blast.cgi

2 https://solgenomics.net/organism/Petunia_axillaris/genome

3 https://david.ncifcrf.gov/

4 https://www.genome.jp/kegg/pathway.html

5 www.ptm-biolab.com.cn
