# Supplementary material for: PhDHS Is Involved in Chloroplast Development in Petunia
Source: Front Plant Sci. 2019 Mar 13;10:284. doi: 10.3389/fpls.2019.00284 (PMC6424912; doi:10.3389/fpls.2019.00284)
Supplement: Supplementary file 1 [file Table_1.doc]

**Title: PhDHS is involved in chloroplast development in petunia**

Juanxu Liu*, Xinlei Chang*, Beibei Ding, Shan Zhong, Li Peng, Qian Wei, Jie Meng, Yixun Yu

**Table S1** Primer sequences for amplification of ***PhDHS*** **and *PhCH42***

| Gene | Forward primer (5′→3′) | Reverse primer (5′→3′) |
| --- | --- | --- |
| *PhDHS* degenerate primers | ATHAARTGYYTNGCNCC | YTCRTCNGGNCKNGCNCC |
| *PhDHS* specific primers | GCCATGAATAGTGAAGCTGTC | CAGTAGTTGTCATTTGGAACC |
| *PhCH42* degenerate primers | GCNATHGTNGGNCARGAYGARATG | NCKYTCYTCNACDATYTTNAC |
| *PhCH42* specific primers | GAGTCATATAAGGAAGAACAAG | CACCCTGTCCTCAGTAGCACC |

**Table S2** Primer sequences of ***PhDHS*,** ***PhCH42, PhPDS* and** *PhACTIN* used in quantitative real-time PCR

| Gene | Forward primer (5′→3′) | Reverse primer (5′→3′) |
| --- | --- | --- |
| *PhDHS* | CATTTCCCATATTAGTAGCT | GTACCCTTTCTAGTCAGTTT |
| *PhCH42* | TGCAGAACTGAATGTTGAT | ATGACAGTGGCAATATCCT |
| *PhPDS* | AAGACGCTCGAAGGCTAA | ATCAGGGCGGAAGAATAA |
| *PheIF5A-1* | GTTCCCTCTTCTCACAAC | AATCCTTACCCTCAGCAA |
| *PheIF5A-2* | AAGCATGGACATGCAAAG | CATGTGGCACATCACAGT |
| *PheIF5A-4* | TTATAGTCATGGCAAAAG | TGAGAAGACTTAAAGGTAG |
| *PhACTIN* | TGCTGATCGAATGAGCAAGGAA | GGAGCAACAACCTTAATCTTC |

**Table S3** Primer sequences of ***PhDHS*** used in subcellular localization

| Gene | Forward primer (5′→3′) | Reverse primer (5′→3′) |
| --- | --- | --- |
| *PhDHS* | AGTCGAGCTCAAATGCTAGATTGGAGGCTTTCA | CGCGGATCCGTCAAACTTGGCACCTTATGT |

**Table S4** Primer sequences of ***PhDHS*,** ***PhCH42* and *PhPDS*** used in VIGS

| Gene | Forward primer (5′→3′) | Reverse primer (5′→3′) |
| --- | --- | --- |
| *PhDHS* (3UTR) | GCTGGATCCTACATTAACACCGCACAA | CCGGAATTCTCTAAACTCAGGGTACTTG |
| *PhDHS*  (5UTR) | GCGGATCCGGAATCAGTAAGATCAGTTGT | CGGAATTCCTTTCTTCTTCACTGCAATCC |
| *PhCH42* | GCGGATCCGTACAGGGAAGTCCACCACA | CGGAATTCAAGAAGACCAGGCTCAAAT |
| *PhPDS* | CGGAATTCTAGTGGAATCGGCAGCAAAGG | GGGGTACCGGGTCCATCCACTCCAAGCTC |

**Table S5** Comparative analysis of PhDHS amino acid sequences with its closest homologues in *Arabidopsis thaliana*, *Saccharomyces* *cerevisiae*, *Drosophila* *melanogaster* and *Homo sapiens*

| Identity | PhDHS | AtDHS | ScDHS | DmDHS |
| --- | --- | --- | --- | --- |
| AtDHS | 74.2% | 100% |  |  |
| ScDHS | 57.0% | 58.1% | 100% |  |
| DmDHS | 54.7% | 58.6% | 55.4% | 100% |
| HsDHS | 59.7% | 60.2% | 60.6% | 61.8% |

**Table S6** Photosynthesis-associated proteins down-regulated in *PhDHS*-silenced plants

PsaA, PsaD, PsaE, PsaF, PsaH, PsaK, PsaL, PsaN and PsaO

| Protein accession | Gene name | *DHS*-s vs CK Ratio | Photosynthesis type |
| --- | --- | --- | --- |
| Peaxi162Scf00485g00109.1 | PetA | 0.167 | Cytochrome b6/F complex |
| Peaxi162Scf00527g00321.1 | PetB | 0.209 | Cytochrome b6/F complex |
| Peaxi162Scf00021g00087.1 | PetC | 0.215 | Cytochrome b6/F complex |
| Peaxi162Scf00174g00103.1 | PetE | 0.301 | Photosynthethic electron transport |
| Peaxi162Scf00328g00108.1 | PetH | 0.629 | Photosynthethic electron transport |
| Peaxi162Scf00160g01617.1 | delta | 0.652 | F-type ATPase |
| Peaxi162Scf00007g00127.1 | PsaC | 0.598 | Photosystem I |
| Peaxi162Scf00642g00013.1 | PsaD1 | 0.29 | Photosystem I |
| Peaxi162Scf00092g00145.1 | PsaD2 | 0.499 | Photosystem I |
| Peaxi162Scf00517g00118.1 | PsaE1 | 0.154 | Photosystem I |
| Peaxi162Scf00043g00067.1 | PsaE2 | 0.324 | Photosystem I |
| Peaxi162Scf00267g01017.1 | PsaF | 0.401 | Photosystem I |
| Peaxi162Scf00045g02242.1 | psaK | 0.44 | Photosystem I |
| Peaxi162Scf01242g00025.1 | PsaL1 | 0.37 | Photosystem I |
| Peaxi162Scf00841g00210.1 | PsaL2 | 0.431 | Photosystem I |
| Peaxi162Scf01257g00019.1 | PsaN | 0.247 | Photosystem I |
| Peaxi162Scf00610g00313.1 | Psb27 | 0.348 | Photosystem II |
| Peaxi162Scf00299g00112.1 | PsbB | 0.274 | Photosystem II |
| Peaxi162Scf00395g00711.1 | PsbC | 0.367 | Photosystem II |
| Peaxi162Scf00060g00176.1 | PsbD | 0.279 | Photosystem II |
| Peaxi162Scf00535g00528.1 | PsbO2A | 0.147 | Photosystem II |
| Peaxi162Scf00589g00517.1 | PsbO2B | 0.2 | Photosystem II |
| Peaxi162Scf00681g00436.1 | psbP2 | 0.389 | Photosystem II |
| Peaxi162Scf00222g00618.1 | PsbQ1 | 0.429 | Photosystem II |
| Peaxi162Scf00082g01216.1 | PsbQ2A | 0.35 | Photosystem II |
| Peaxi162Scf00257g00046.1 | PsbQ2B | 0.517 | Photosystem II |
| Peaxi162Scf00232g01129.1 | PsbR | 0.234 | Photosystem II |
| Peaxi162Scf00156g00139.1 | Lhca1 | 0.609 | LHC |
| Peaxi162Scf00906g00019.1 | Lhca3 | 0.229 | LHC |
| Peaxi162Scf00000g51016.1 | Lhcb1 | 0.236 | LHC |
| Peaxi162Scf00141g00022.1 | Lhcb4.1 | 0.281 | LHC |
| Peaxi162Scf00009g01816.1 | Lhcb4.1 | 0.287 | LHC |
| Peaxi162Scf00402g00937.1 | Lhcb5-2 | 0.487 | LHC |
| Peaxi162Scf00152g01119.1 | Lhcb6 | 0.192 | LHC |

**Table S7** Thylakoid -associated proteins down-regulated in *PhDHS*-silenced plants

| Protein accession | Gene description | DHS-s vs CK Ratio | Photosynthesis type |
| --- | --- | --- | --- |
| Peaxi162Scf00434g00329.1 | Thylakoid lumen 18.3 kDa protein | 0.419 | thylakoid |
| Peaxi162Scf00736g00213.1 | Thylakoid lumenal 15 kDa protein 1, chloroplastic | 0.262 | thylakoid |
| Peaxi162Scf00311g00059.1 | Thylakoid lumenal 15.0 kDa protein 2 | 0.371 | thylakoid |
| Peaxi162Scf00313g00815.1 | Thylakoid lumenal 16.5 kDa protein, chloroplastic | 0.663 | thylakoid |
| Peaxi162Scf00089g00121.1 | Thylakoid lumenal 17.9 kDa protein | 0.423 | thylakoid |
| Peaxi162Scf00428g00063.1 | Thylakoid lumenal 19 kDa protein, chloroplastic | 0.217 | thylakoid |
| Peaxi162Scf00030g02222.1 | Thylakoid lumenal 29 kDa protein, chloroplastic | 0.618 | thylakoid |
| Peaxi162Scf00002g00075.1 | Thylakoid lumenal protein | 0.248 | thylakoid |
| Peaxi162Scf01058g00001.1 | Thylakoid soluble phosphoprotein | 0.129 | thylakoid |
| Peaxi162Scf00007g02516.1 | Protein thylakoid formation1, chloroplastic | 1.259 | thylakoid |
| Peaxi162Scf00417g01337.1 | Chloroplast thylakoid membrane | 0.36 | thylakoid |
| Peaxi162Scf00313g00019.1 | Thylakoid membrane phosphoprotein 14 kDa | 0.68 | thylakoid |


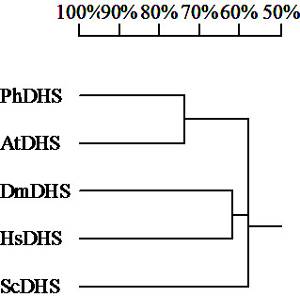


A

B

**Figure S1** Predicted amino acid sequence alignments and neighbour-joining trees of DHS. A, Predicted amino acid sequence alignments of PhDHS with *Arabidopsis* AtDHS (NP_196211), yeast ScDHS (EGA74636), *Drosophila melanogaster* DmDHS (NP_648188) and *Homo sapiens* HsDHS (AAA96151). Conserved residues are shaded in black. Grey shading indicates similar residues in three out of five of the sequences. Underlined amino acids refer to the spermidine-binding site (236-349). Underscore refers to the NAD-binding site from serine 97 to aspartic acid 349. The active centre of the DHS protein from glutamine 330 to lysine 335 is highlighted in the black box. B, Neighbour-joining trees among proteins encoded by the DHS-like genes using DNAMAN.

pTRV2

pTRV2-PhDHS

α-PhDHS

α-Actin

*PhDHS*

Relative expression levels

*

pTRV2

pTRV2-PhDHS

A

B


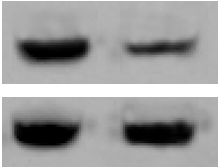


**Figure S2** The effects of pTRV2-PhDHS treatment on the expression of PhDHS in the fifth leaves under the buds from seven-week-old plants. A, *PhDHS* mRNA level as determined by quantitative real-time PCR. Petunia *Actin* (accession no. FN014209) served as an internal reference gene. The relative expression levels are shown as fold change values. The data are presented as the mean ± SD (n = 3). B, *PhDHS* protein level as determined by western blotting with Actin as internal reference.


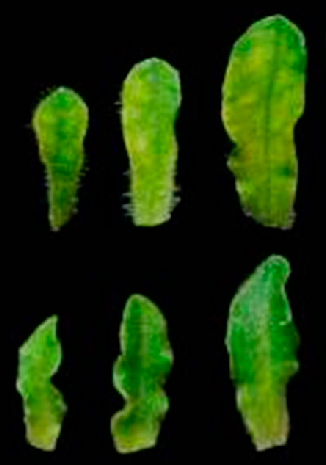


**Figure S3** Effect of *PhDHS* silencing on the sepals in petunia. Up, Sepals in *PhDHS*-silenced plants; down, Sepals in pTRV2 empty vector-infected plants. Scale bar, 0.8 cm.

*PhCH42*

*PhPDS*

A

B

Relative expression levels

*

*

pTRV2

pTRV2-PhCH42

pTRV2-PhPDS

pTRV2

**Figure S4** The effects of pTRV-PhCH42 (A) and pTRV-PhPDS (B) treatment on the expression of *PhCH42* and *PhPDS* in the fifth leaves under the buds from seven-week-old plants as determined by quantitative real-time PCR, respectively. Petunia *Actin* (accession no. FN014209) served as an internal reference gene. The relative expression levels are shown as fold change values. The data are presented as the mean ± SD (n = 3).

Fv/Fm

pTRV2

pTRV2-PhDHS

*

**Figure S5** Effects of *PhDHS* silencing on the maximum quantum yield of PSII photochemistry (Fv/Fm) in petunia [leaves](https://www.sciencedirect.com/topics/agricultural-and-biological-sciences/leaves). Data represent the means of independent measurements of 5 replicates with standard deviations shown by vertical error bars. Asterisk means significant difference at P = 0. 05 level.

pTRV2

pTRV2-PhDHS


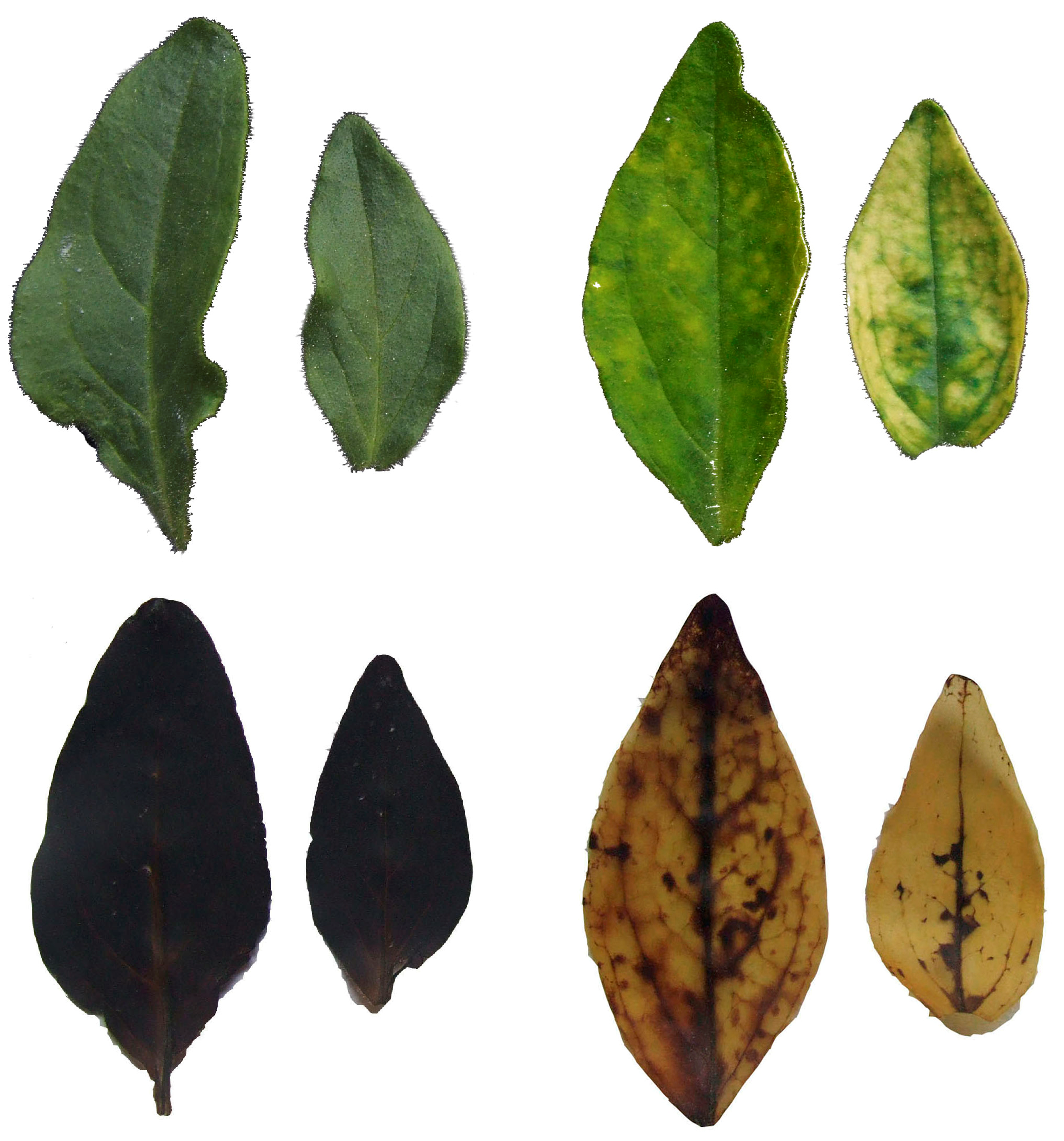


**Figure S6** Iodine staining of 5-week-old mature and young leaves in pTRV2 empty vector-infected (left) and *PhDHS*-silenced plants (right). Up, the leaves before staining; down, the leaves after staining.


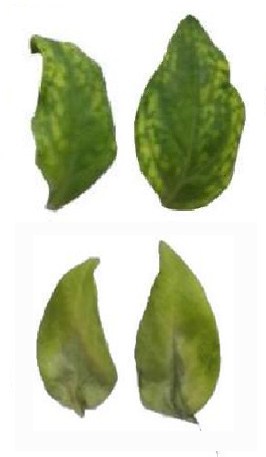

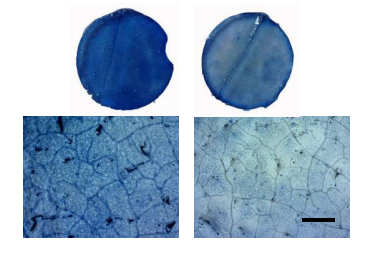


A

B

**Figure S7** *PhDHS* silencing delays leaf senescence. A, mature leaves of pTRV2 empty vector-infected plants (down) and *PhDHS*-silenced plants (up) at 10 day after harvest. B, Trypan blue–stained leaves disc (up) and macroscopic phenotypes (down) from pTRV2-infected (left) and *PhDHS*-silenced plants (right) on 10 day after harvest. Scale bar, 150 μm.


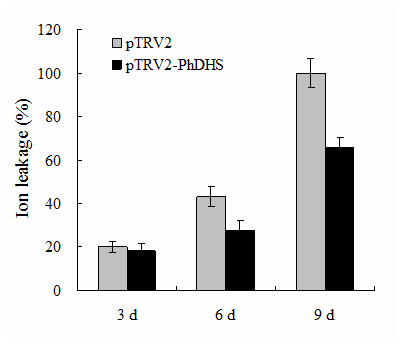


*

*

**Figure S8** Ion leakage rate of the sixth leaf tissues of plants 5 weeks after infection. Data are mean ± SD from three biological replicates. Asterisk means signifcant diference at P = 0. 05 level.


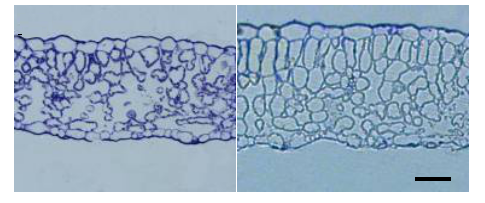


**Figure S9** Section cross of leaves in pTRV2-infected plants (control) (left) and *PhDHS*-silenced plants (right). Bar= 20 µm.


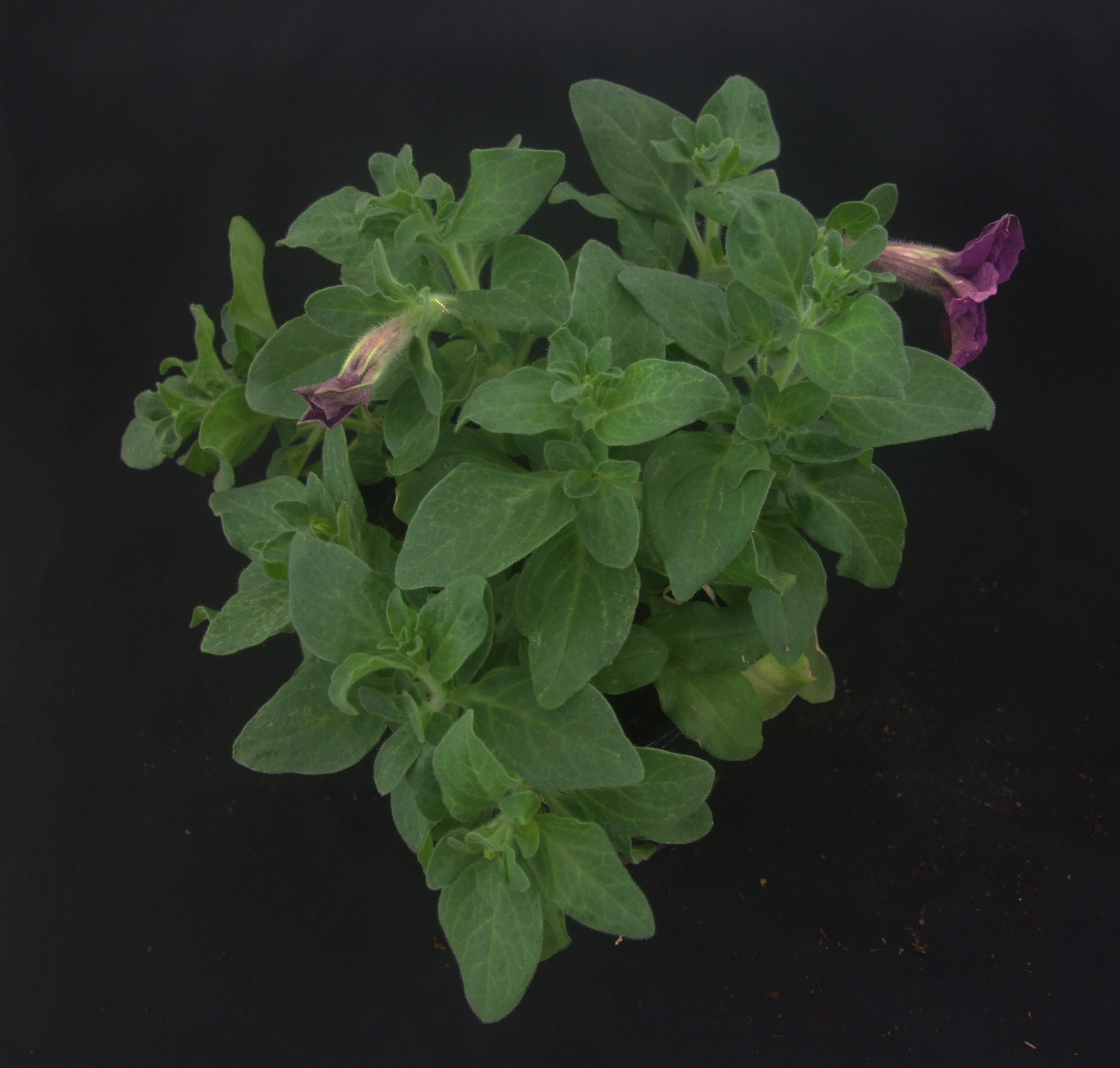

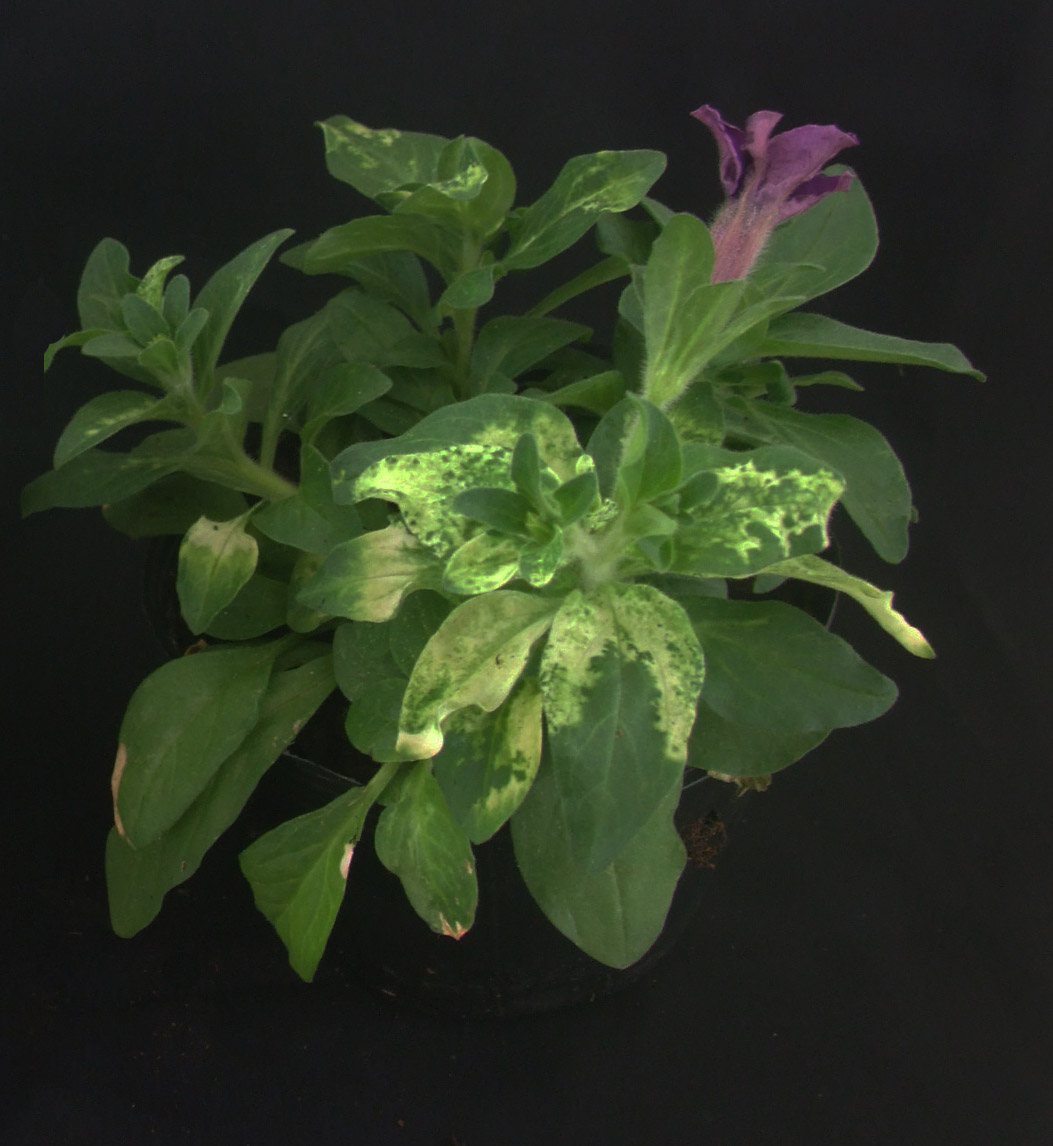

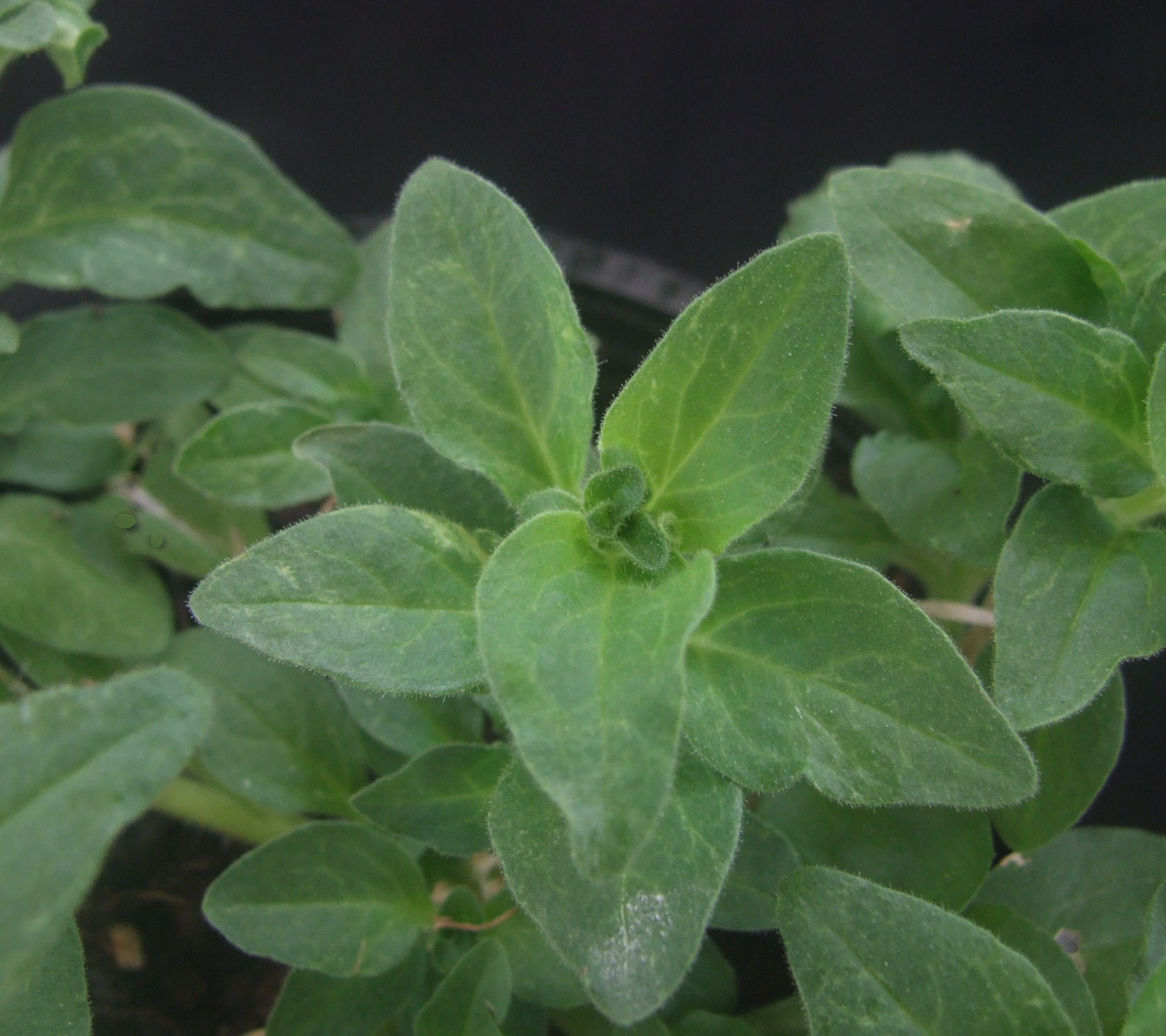

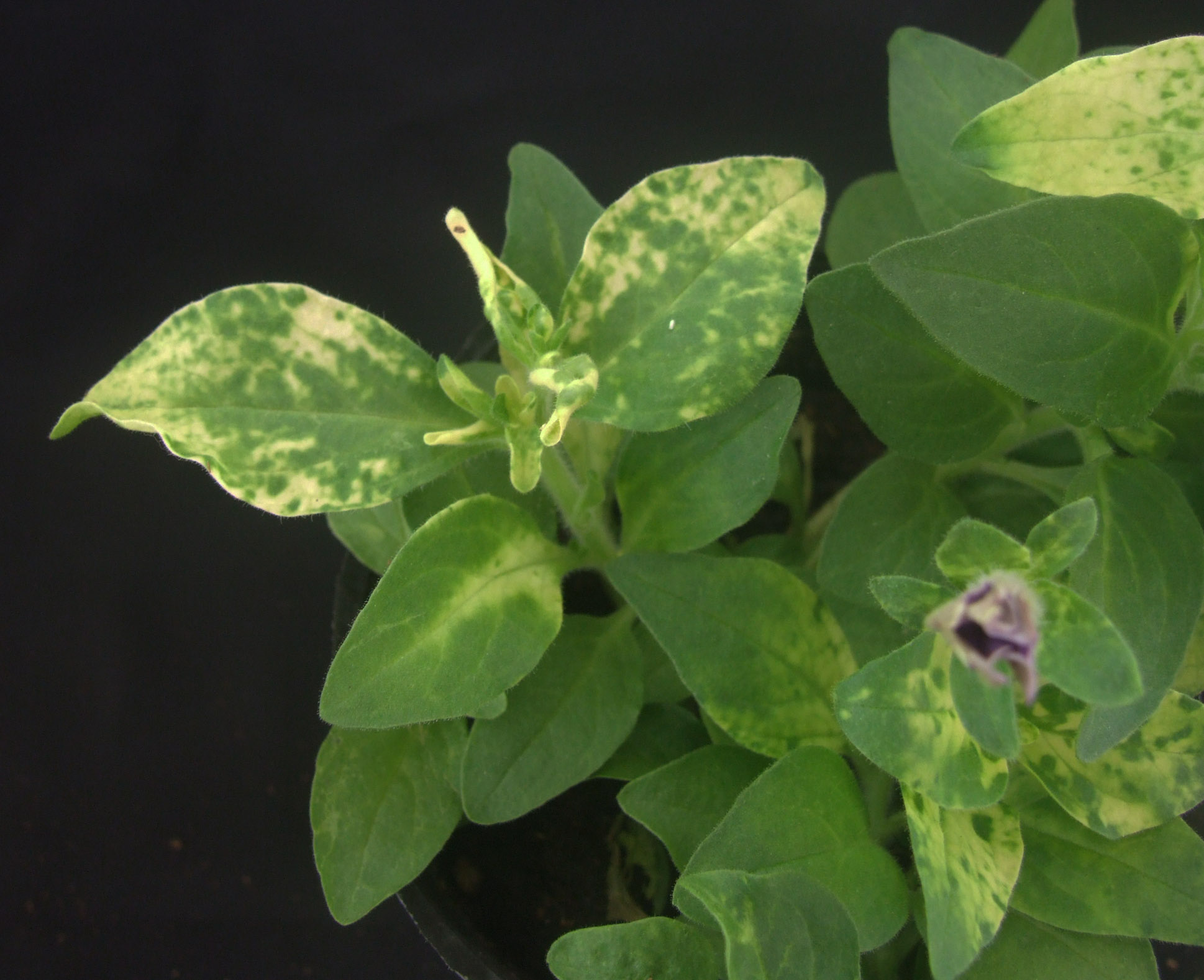

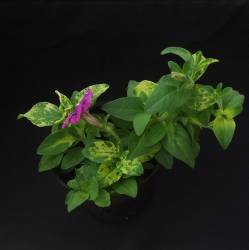

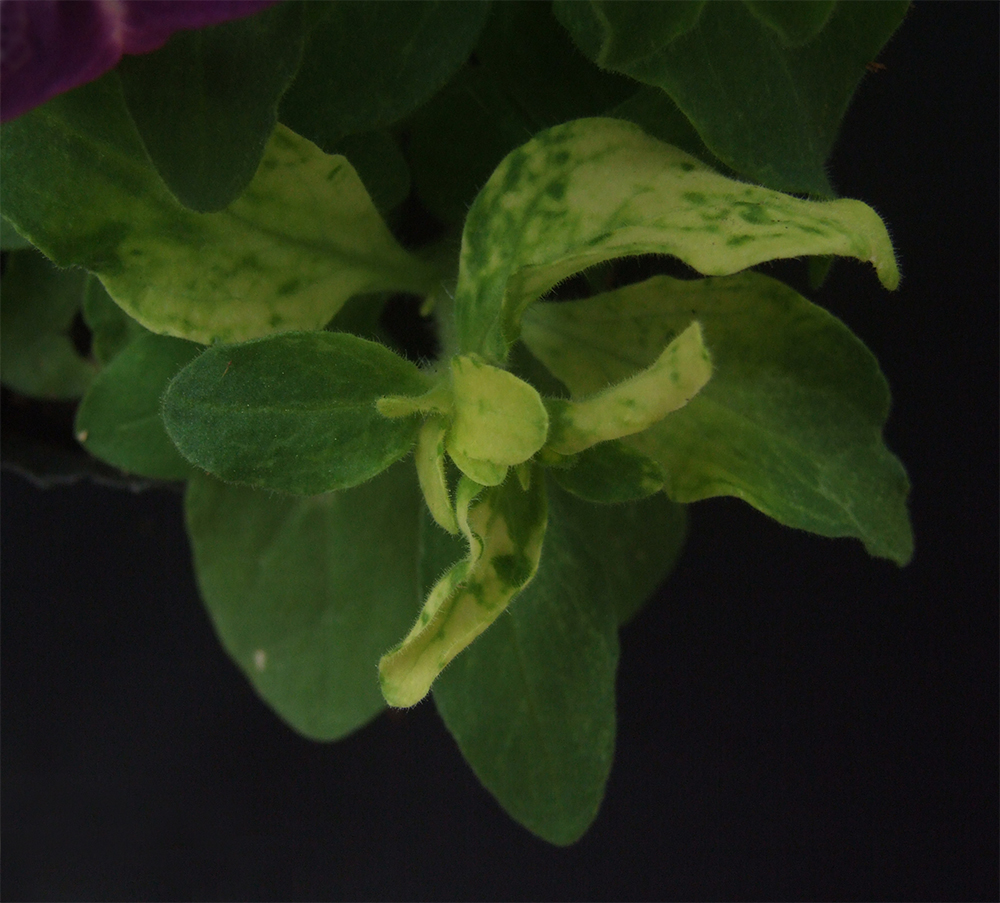

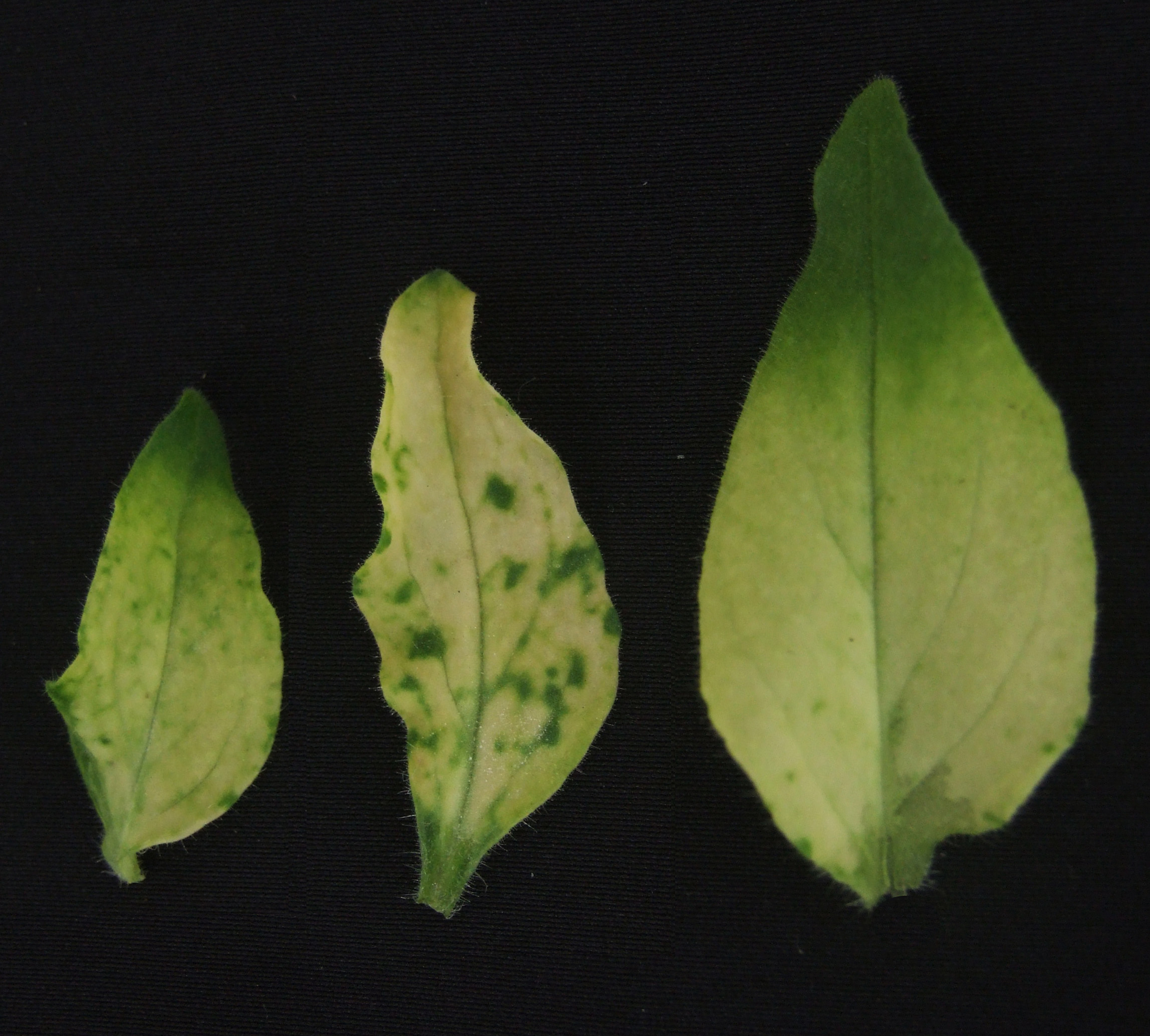

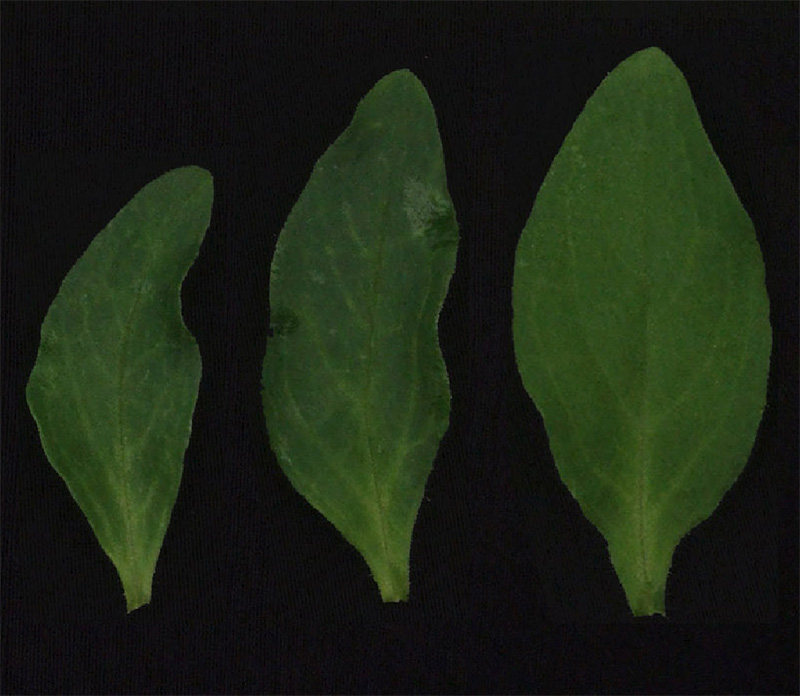


pTRV2

pTRV2-PhDHS

pTRV2-PhDHS-

5UTR

A

I

D

E

G

F

C

B

H

**Figure S10** Phenotype of pTRV2-, pTRV2-PhDHS- and pTRV2-PhDHS-5UTR-infected plants. Seven-week-old plants exhibiting different leaf color in pTRV2-(A-C), pTRV2-PhDHS-(D-F) and pTRV2-PhDHS-5UTR-(G-I) infected plants. A, D, G, Scale bar, 8.0 cm. B, E, H, Scale bar, 1.8 cm. C, F, I, Scale bar, 1.3 cm.

Relative expression levels

pTRV2

pTRV2-PhDHS-

5UTR

*PhDHS*

**Figure S11** The effects of pTRV2-PhDHS-5UTR treatment on the expression of *PhDHS* in the fifth leaves under the buds from seven-week-old plants as determined by quantitative real-time PCR. Petunia *Actin* (accession no. FN014209) served as an internal reference gene. The relative expression levels are shown as fold change values. The data are presented as the mean ± SD (n = 3).

A

B


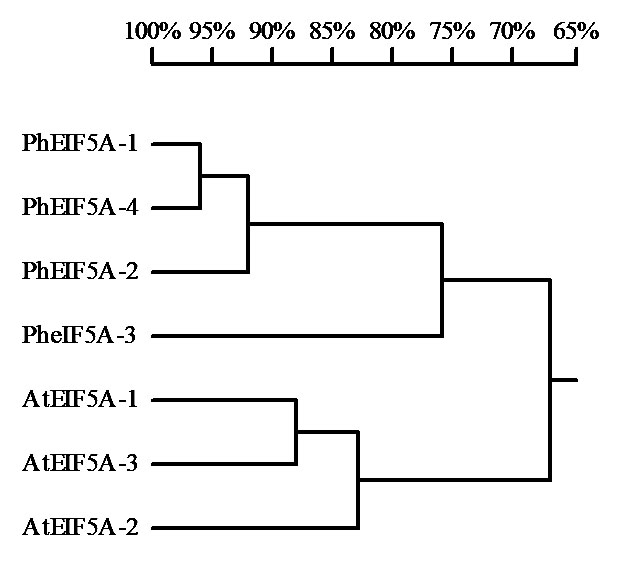


**Figure S12** Predicted amino acid sequence alignments and neighbour-joining trees of eIF5A. A, Predicted amino acid sequence alignments of four PheIF5As with *Arabidopsis* *thaliana* AteIF5A-1 (At1g13950), AteIF5A-2 (At1g26630) and AteIF5A-3 (At1g69410). Conserved residues are shaded in black. Grey shading indicates similar residues in three out of five of the sequences. B, Neighbour-joining trees among proteins encoded by the eIF5A-like genes using DNAMAN.

Relative expression levels

R

S

L

C

R

S

L

C

R

S

L

C

a

b

c

d

c

c

b

a

a

b

b

b

*PheIF5A1*

*PheIF5A2*

*PheIF5A4*

A

B

C

**Figure S13** The expression patterns of three *PheIF5A* genes determined by quantitative real-time PCR in different organs. Petunia *Actin* (accession no. FN014209) served as an internal reference gene. R, roots; L, leaves; S, stems; C, corollas. The relative expression levels are presented as fold-change values. The data are presented as the mean ± SD (n = 3). Different letters mean significant differences at the P=0.05 level.

**Figure S14** Reproducibility analysis of 3 repeated trials based on Pearson correlation coefficient.


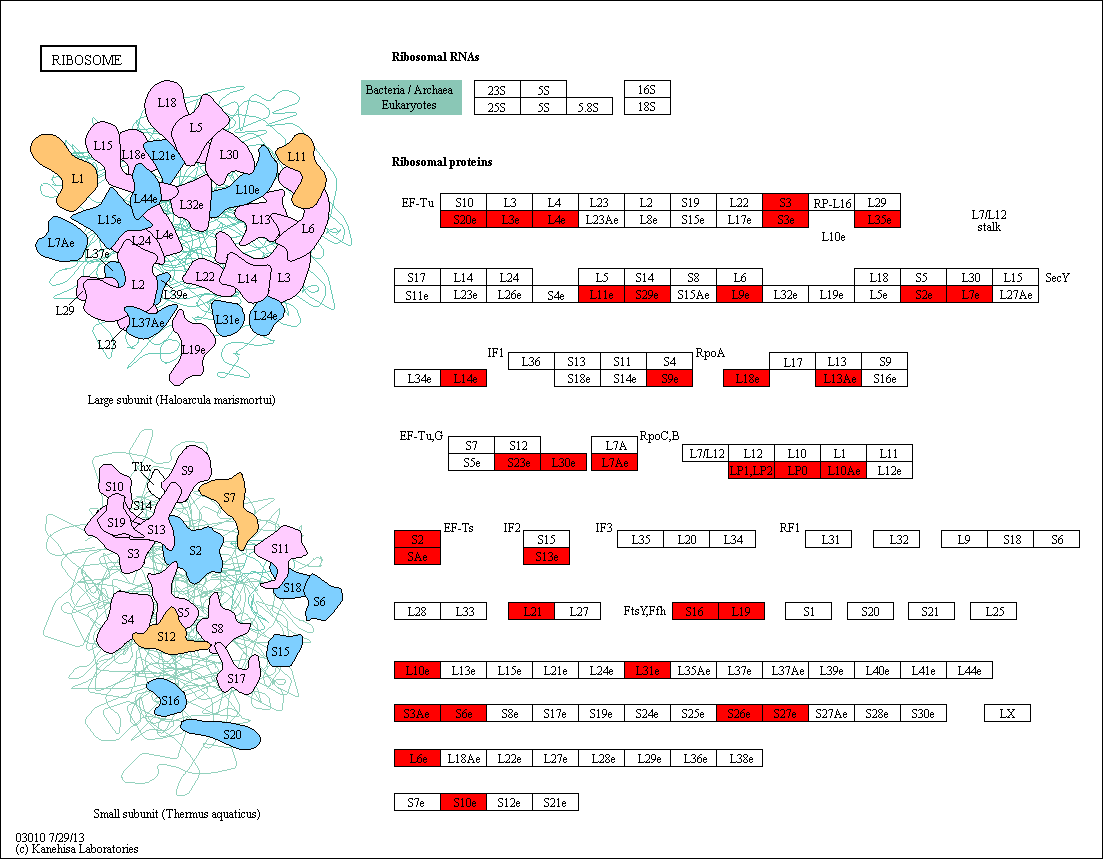


**Figure S15** Effects of *PhDHS* silencing on Ribosome - Reference pathway (KEGG Pathway: sly03010) in petunia. The red box indicates up-regulation at the protein level in *PhDHS*-silenced plants compared with control.


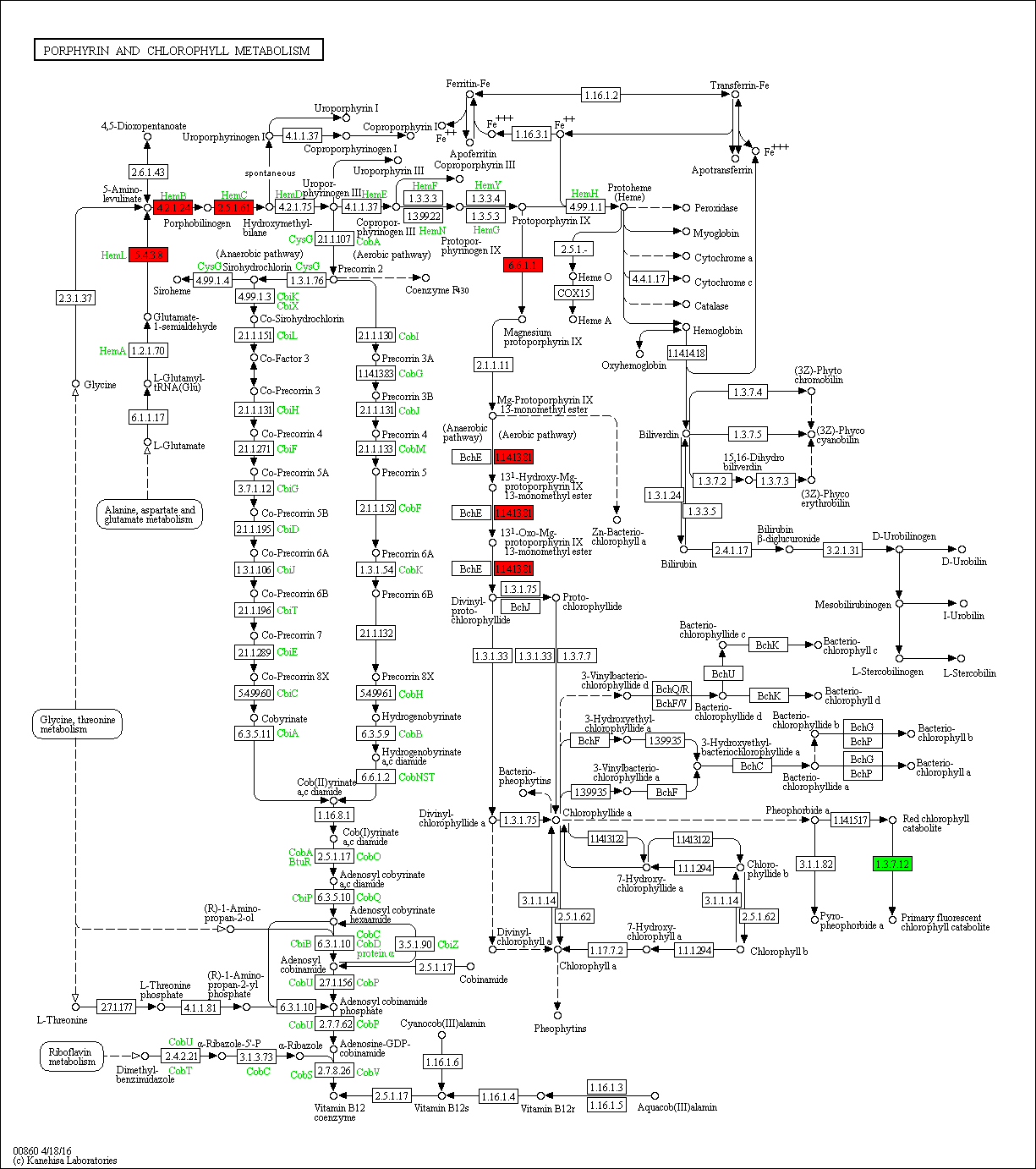


**Figure S16** Effects of *PhDHS* silencing on the porphyrin and chlorophyll metabolism pathway (KEGG Pathway: sly00860) in petunia. The red box indicates up-regulation, and the green box indicates down-regulation at the protein level in *PhDHS*-silenced plants compared with control.


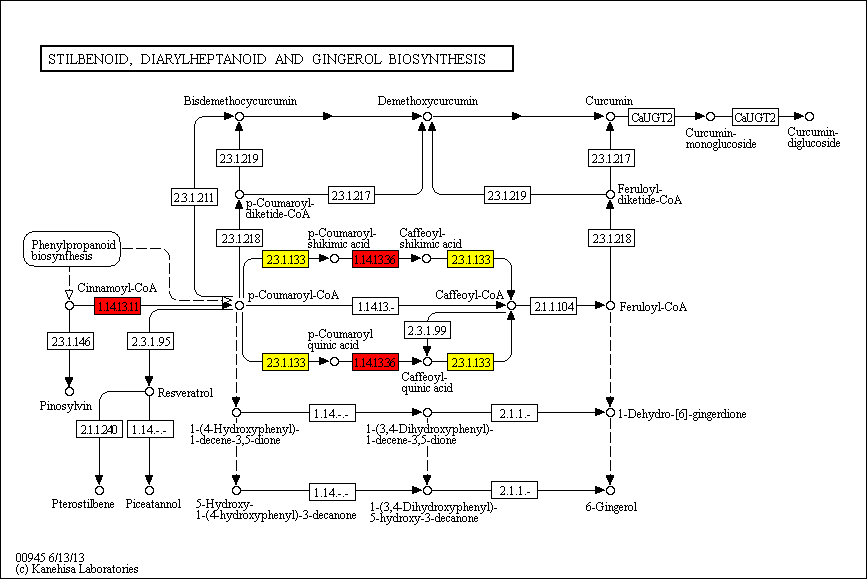


**Figure S17** Effects of *PhDHS* silencing on the Stilbenoid, diarylheptanoid and gingerol biosynthesis pathway (KEGG Pathway: sly00945) in petunia. The red box indicates up-regulation and the yellow box indicates both down- and up-regulation at the protein level in *PhDHS*-silenced plants compared with control.


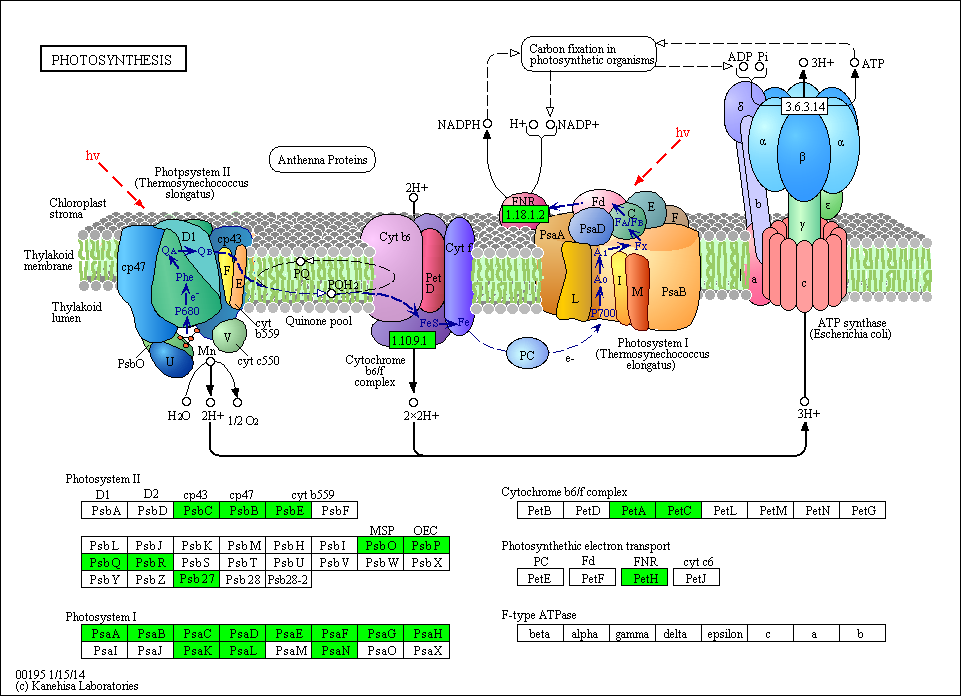


**Figure S18** Effects of *PhDHS* silencing on Photosynthesis - Reference pathway (KEGG Pathway: sly00195) in petunia. The red box indicates up-regulation, and the green box indicates down-regulation at the protein level in *PhDHS*-silenced plants compared with control.


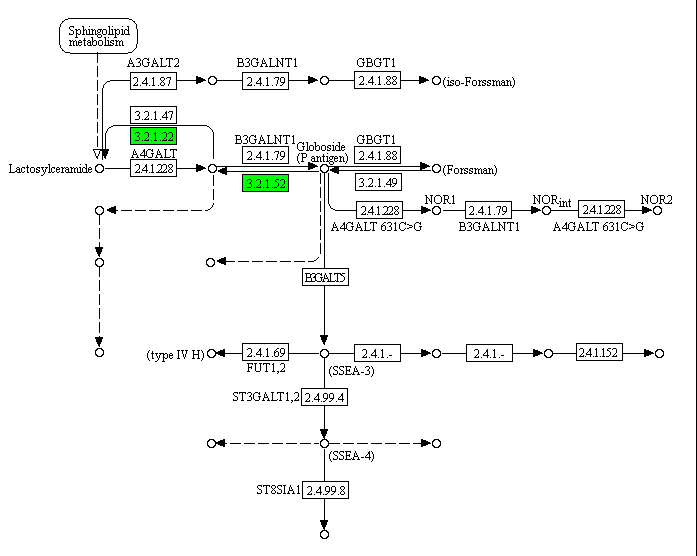
**Figure S19** Effects of *PhDHS* silencing on Sphingolipid metabolism pathway (KEGG Pathway: sly00603) in petunia. The the green box indicates down-regulation at the protein level in *PhDHS*-silenced plants compared with control.


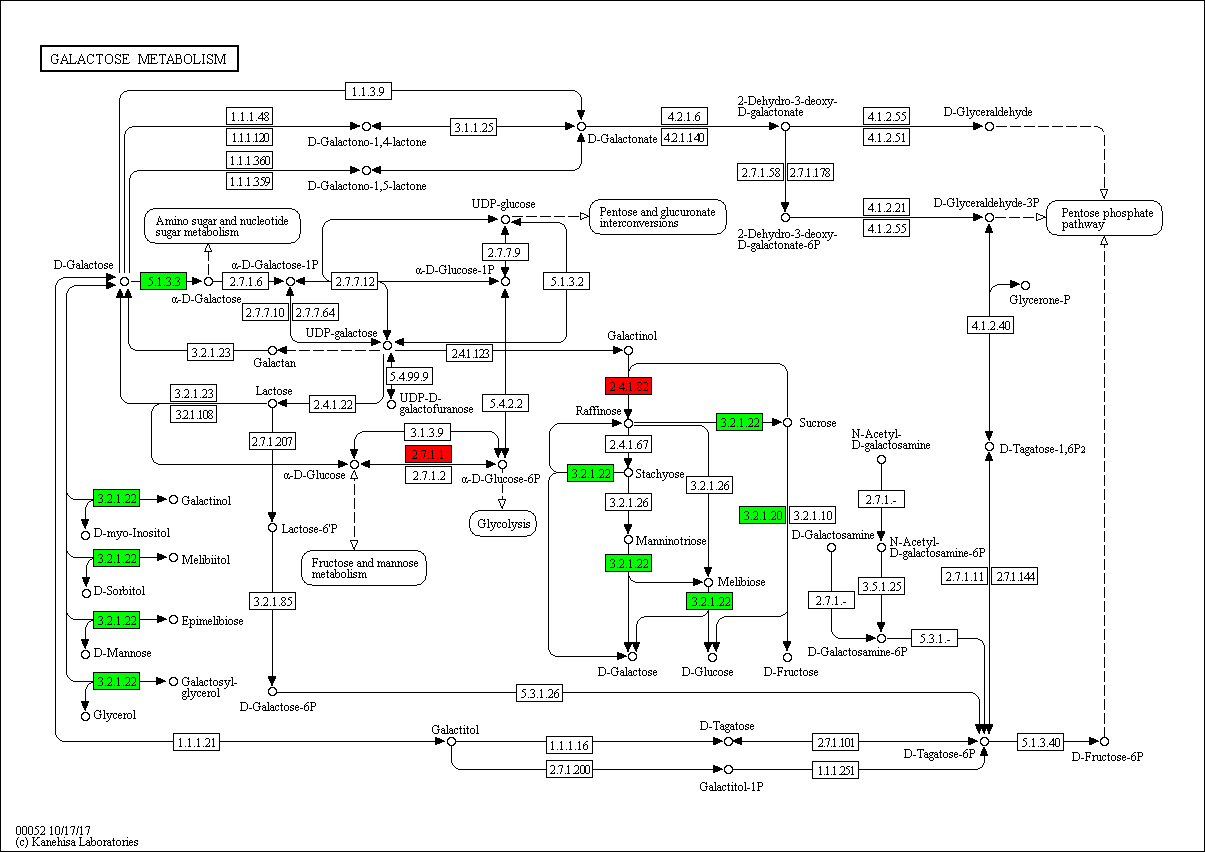


**Figure S20** Effects of *PhDHS* silencing on Galactose metabolism Reference pathway (KEGG Pathway: sly00052) in petunia. The red box indicates up-regulation, and the green box indicates down-regulation at the protein level in *PhDHS*-silenced plants compared with control.



α-β-Actin

α-PsbD

α-PsbQ


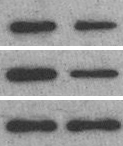


pTRV2

pTRV2-

PhDHS

**Figure S21** Confirmation of proteome data by western blotting. Western blotting analysis of PsbD and PsbQ in petunia leaves treated with pTRV2 or pTRV2-PhDHS vector. The representative results from three independent experiments were indicated.
